# Photolithographic Microfabrication of Microbatteries for On-Chip Energy Storage

**DOI:** 10.1007/s40820-024-01625-9

**Published:** 2025-01-08

**Authors:** Yuan Ma, Sen Wang, Zhong-Shuai Wu

**Affiliations:** 1 State Key Laboratory of Catalysis, Dalian Institute of Chemical Physics, Chinese Academy of Sciences, Dalian, 116023 People’s Republic of China; 2 School of Transportation Engineering, Dalian Jiaotong University, Dalian, 116028 People’s Republic of China; 3 University of Chinese Academy of Sciences, Beijing, 100049 People’s Republic of China; 4 Dalian National Laboratory for Clean Energy, Chinese Academy of Sciences, Dalian, 116023 People’s Republic of China

**Keywords:** Microbatteries, Photolithography, Internet of Things, Micropatterns, On-chip energy storage

## Abstract

Microbatteries (MBs) are crucial to power miniaturized devices for the Internet of Things. In the evolutionary journey of MBs, fabrication technology emerges as the cornerstone, guiding the intricacies of their configuration designs, ensuring precision, and facilitating scalability for mass production. Photolithography stands out as an ideal technology, leveraging its unparalleled resolution, exceptional design flexibility, and entrenched position within the mature semiconductor industry. However, comprehensive reviews on its application in MB development remain scarce. This review aims to bridge that gap by thoroughly assessing the recent status and promising prospects of photolithographic microfabrication for MBs. Firstly, we delve into the fundamental principles and step-by-step procedures of photolithography, offering a nuanced understanding of its operational mechanisms and the criteria for photoresist selection. Subsequently, we highlighted the specific roles of photolithography in the fabrication of MBs, including its utilization as a template for creating miniaturized micropatterns, a protective layer during the etching process, a mold for soft lithography, a constituent of MB active component, and a sacrificial layer in the construction of micro-Swiss-roll structure. Finally, the review concludes with a summary of the key challenges and future perspectives of MBs fabricated by photolithography, providing comprehensive insights and sparking research inspiration in this field.

## Highlights

- The fundamental principles and step-by-step procedures of photolithography are introduced, and a nuanced understanding of its operational mechanisms and the criteria for photoresist selection is offered.
- Various specific roles that photolithography plays in microbatteries (MBs) fabrication, including templates for 2D and 3D micropatterns, MB active components, and the sacrificial layer for constructing micro-Swiss-roll structure, are elaborated.
- The challenges and future directions of MBs fabricated using photolithography, including materials selection, packaging techniques, application, and performance evaluation, are discussed.

## Graphical abstract

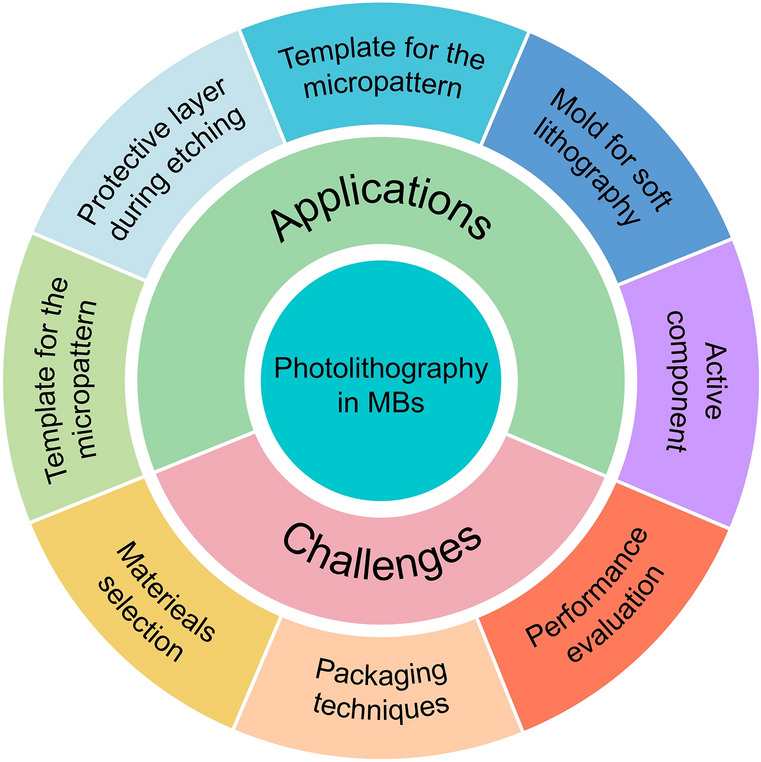

## Introduction

With the continuous development of microelectronics and microsystems, the Internet of Things (IoT) era characterized by digitalization, intelligence, and the “interconnection of all things” is coming [1–6]. Miniaturized electronic devices (MEDs) are significant parts of IoT, including micro-electro-mechanical systems, microsensors, intelligent electronic equipment, micro-robots, and implantable medical devices [7]. Complex tasks containing signal detection, information processing, mechanical actuation, and wireless communications can be accomplished by such MEDs, and the MEDs can be distributed everywhere to connect everything by their ultra-small size. Places inaccessible to traditional electronic devices, such as narrow areas inside machines, leaves or roots of crops, or even blood vessels inside the human body, can be arranged with MEDs [8]. By sensing various surrounding signals, intelligently analyzing and processing, and sending the data to the database by wireless transmission, MEDs can conveniently monitor the environment, agriculture, and medical health. Such an invisible net could connect everything, achieving the IoT and facilitating more efficient decision-making [5, 9]. Critically, the power supply serves as the cornerstone, enabling the functionality of all these MEDs and ensuring their seamless operation [10]. Although various kinds of energy from the environment can be harvested by solar cells, thermo-electric devices, and nanogenerators, these forms of energy are inherently unstable and discontinuous, hindering the steady operation of the MEDs [10]. Consequently, electrochemical energy storage devices such as batteries, with high energy density achieving continuous energy supply, are indispensable [9, 11–14]. Given that the size of MED is usually at the millimeter or even micron level, the batteries employed in these applications, also known as microbatteries (MBs), must possess ultra-small dimensions, customizable shapes, facile integration, and high energy density to meet the requirements of MEDs [6, 11, 14]. Regrettably, miniaturizing traditional batteries into MBs introduces challenges and more stringent criteria regarding configuration design, microfabrication technology, and material selection [10]. The configuration of traditional batteries, such as coin-type, cylinder-type, pouch-type, and prismatic-type, involves stacking or winding the cathode, anode, and separator together [15–17]. However, due to the tiny size and high precision requirements, the stacking and winding technology may not be suitable for manufacturing MBs [18, 19]. Besides, MBs often require more complex and precise configuration designs to ensure optimal performance. For instance, some MBs utilize interdigital and 3D configurations to maximize energy density within a small space. These configurations can only be created using advanced microfabrication techniques, which allow for the precise patterning and structuring of battery components [1, 2, 4, 11, 12, 20–22]. Furthermore, the miniaturization and unique configuration of MBs do present significant challenges in terms of electrode slurry coating and electrolyte injection processes traditionally employed in battery manufacturing [23], as there is a risk of short circuits, and it is also difficult to confine the electrolyte within such a small space. Advanced microfabrication technologies are highly required to load the electrode and electrolyte precisely to ensure the successful manufacture of MBs without compromising their performance and safety. Accordingly, the electrode and the electrolyte materials used for MBs should be stable and durable under the conditions of the microfabrication processes, in addition to delivering high energy and power densities, which imposes significant restrictions on the choice of materials [10].

Fortunately, a variety of advanced microfabrication technologies have been developed to fabricate MBs, including photolithography, laser scribing [24–35], mask-assisted filtration [36–42], screen printing [43–46], stencil printing [47, 48], spray coating [49], electrohydrodynamic jet printing [50], and 3D printing [51–57] (Table 1). Photolithography stands out as a technology that possesses a high resolution at the micrometer or even nanoscale, which is crucial for the configuration design of MBs applied in the IoTs. Complex designs (e.g., 3D electrode architectures [18, 19, 58–65]) that provide higher energy density and power density for the MBs can also be obtained by photolithography. Meanwhile, the widespread application of photolithography in the semiconductor industry further underscores its good compatibility with the subsequent MEDs processing, facilitating seamless integration and optimization [3]. In addition, photolithography is a parallel process that makes it possible to produce numerous tiny devices simultaneously, giving it an advantage in customizable energy supply [66, 67]. When photolithography is combined with other microfabrication process technologies (such as etching and thin film deposition), it will exhibit even more diversified functions, enabling the mass production of MBs [18, 19, 65, 68]. To sum up, photolithography is an ideal technology for fabricating MBs. However, comprehensive reviews on the photolithographic microfabrication of MBs are still scarce. Herein, we will review the recent status and prospects of photolithographic microfabrication for MBs. Firstly, we delve into the principle and procedure of photolithography, providing a clear understanding of its operation and the choice of photoresist. This foundational knowledge serves as a springboard for exploring the intricate applications of photolithography in MB fabrication. Following that, we discuss elaborately specific photolithography applications in MBs, including the template for the micropattern, protective layer during etching, mold for soft lithography, MB active component, and sacrificial layer in micro-Swiss-roll. Each of these applications underscores the versatility and indispensability of photolithography in MB fabrication. Finally, we consolidate our discussion by summarizing the key challenges that currently hinder the widespread adoption of photolithography for MB fabrication. Recognizing these challenges, we offer intriguing perspectives on potential solutions and future directions, aiming to inspire further research and innovation in this burgeoning field. Our goal is to offer a comprehensive overview that highlights the current state of photolithographic microfabrication of MBs and paves the way for future advancements in this field.

**Table 1:** Comparison of various microfabrication technologies for fabricating MBs

Microfabrication technologies	Advantages	Disadvantages
Photolithography	High resolution, excellent process compatibility with electronic devices, convenient mass production	Complicated and time-consuming processing, high limitation of compatible electrode materials, high cost
Laser scribing	High resolution, convenient pattern design	Potential damage to materials, high restriction of material, high cost
Mask-assisted filtration	Simple processing, excellent compatibility with different electrode materials, low cost	Time-consuming processing, low resolution, inconvenient to design complex patterns, high restriction of the substrate
Screen printing	Fast processing, convenient large-scale integration, high material applicability, excellent compatibility with different substrates, low cost	Low resolution and electrode thickness
Stencil printing	Fast processing, convenient large-scale integration, high material applicability, high electrode thickness, low cost	Low resolution
Spray coating	Convenient mass production and large-scale integration, low cost	Low efficiency and electrode thickness, high limitation of materials
Electrohydrodynamic jet printing	High resolution, convenient pattern design	Stringent requirement of appropriate rheological property, high limitation of material, intricate processing parameters
3D printing	High thickness and mass loading of electrode materials, convenient pattern design, excellent compatibility with different electrode materials	Stringent requirement of appropriate rheological property, low electrode bulk density

## Procedure and Principle of Photolithography

**Fig. 1:**
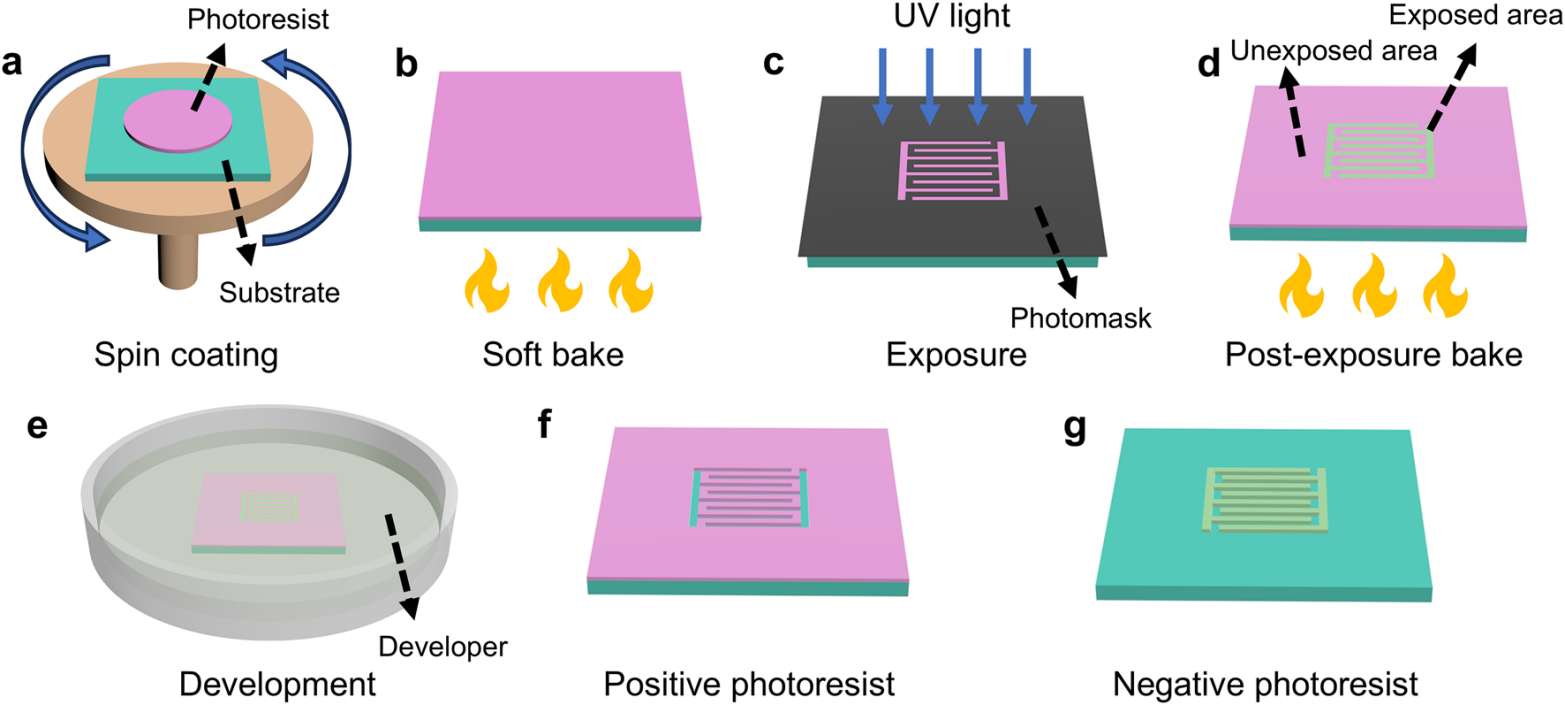
Schematic illustration of the procedure of photolithography. **a** Spin coating of photoresist. **b** Soft bake of photoresist. **c** Exposure of photoresist covered by photomask under UV light. **d** Post-exposure bake of photoresist. **e** Development of photoresist in the developer. **f** The resulting positive photoresist pattern leaving the unexposed area remained. **g** The resulting negative photoresist pattern leaving the exposed area remained

Photolithography is a pivotal process in semiconductor manufacturing, which involves precisely transferring geometric patterns from the photomask onto a photoresist on the substrate surface [69]. It can realize the construction of micropatterns with ultra-high precision at the micron or even nanometer level, which is particularly beneficial for MBs. The photolithography process discussed here is commonly used in general laboratories to prepare MBs, and its resolution is sufficient at the micron level. The photolithography procedure includes substrate pretreatment, spin-coating of photoresist, soft bake, exposure, post-exposure bake, and development (Fig. 1). Firstly, to remove surface contaminants and enhance the adhesion with photoresist, the substrate, such as Si and glass, should be cleaned, pretreated, and dried. Various cleaning methods are widely utilized, including chemical cleaning (acidic, alkaline, and oxidizing cleaning), physical cleaning (ultrasonic and plasma cleaning), and combinations of multiple methods. For example, the substrate is usually cleaned with the piranha solution (3:1 vol% mixture of concentrated H_2_SO_4_ and 30% H_2_O_2_) to remove organic residues, followed by a rinse with deionized water. According to the desired surface properties, reactive ion etching (RIE), O_2_ plasma, and aqueous ammonia can be utilized to increase the hydrophilic of the substrate surface by introducing polar hydrophilic functional groups (e.g., –OH, –COOH, –NH_2_, etcetera). In contrast, hexamethyldisilazane can be selected to modify the hydrophobicity of the substrate surface by introducing hydrophobic trimethylsilyl groups. Subsequently, an appropriate amount of photoresist is spin-coated onto the substrate (Fig. 1a) and followed by a bake process (soft bake) (Fig. 1b). During spin-coating, the photoresist will move to the periphery of the substrate due to the centrifugal force, which will cause uneven distribution of photoresist thickness and produce strain inside the photoresist. The purpose of soft bake is to volatilize the solvent in the photoresist, further homogenize the thickness, and solidify the photoresist, facilitating the following processing. Afterward, the photoresist film is covered by a photomask with a specific pattern and exposed to ultraviolet (UV) light for the photochemical reaction (Fig. 1c), followed by the post-exposure bake step (Fig. 1d) and the development process in a developer (Fig. 1e) to obtain the patterned structure. According to the chemical composition and the type of photochemical reaction, the photoresist can be divided into positive and negative types [70]. In the case of positive photoresist, it contains resin and a photoactive compound that can inhibit the dissolution of resin in the developer. The photoactive compound will decompose under UV light, resulting in the photoresist in the exposed region being selectively dissolved by the developer. Thus, the final pattern of positive photoresist is identical to the photomask. This is also the origin of the name “positive” (Fig. 1f). The negative photoresist contains the resin monomer and the photo-initiator. Under exposure to UV light, the photo-initiator will decompose into free radicals or cations, which can induce the polymerization of monomers within the exposed region during the post-exposure bake process. As a result, the solubility of the exposed part is reduced, and the unexposed region will be selectively dissolved in the developer. Thus, the final pattern of negative photoresist is complementary to the photomask. This is also the origin of the name “negative” (Fig. 1g). The selection of positive or negative photoresists depends on their properties and application requirements. For the positive photoresist, the decomposition reaction of the photoactive compound that occurred in the exposed region will not extend to the unexposed region. Hence, the edge of the final pattern is clear, and the resolution is high. In contrast, the cross-linking reaction in the exposed region of the negative photoresist may extend to a certain extent in the unexposed region, so some molecules in the unexposed region may also be cross-linked, resulting in blurred edges and reduced resolution. Besides, the remaining region of the positive photoresist (the unexposed region) is inert to the developer. It does not absorb the developer during the development process so that the pattern can be better retained and the resolution is higher. On the contrary, the remaining region of the negative photoresist (the exposed region) is the polymer after the cross-linking, which may absorb the developer to a certain extent during the development process and expand, resulting in the distortion of the pattern and the reduction of resolution. In addition, the positive photoresist usually possesses smaller viscosity (resulting in thinner thickness) and is easier to remove, while the negative photoresist usually has greater viscosity (resulting in thicker thickness) and stronger stiffness, so the positive photoresist is often used to define high-precision micropatterns and in scenarios where mild removal is required after using, and the negative photoresist is mainly used for preparing microarchitectures. Exposure and development are the two most significant processes in the photolithography process, and the parameters such as exposure energy, focus, developer amount, and development time need to be strictly controlled, which will determine the size and resolution of the final obtained patterns. Photolithography can realize the construction of micropatterns with ultra-high precision at the micron or even nanometer level, which is particularly beneficial for patterning MBs. However, this precision comes at a cost, as it decreases the active component ratio in the device, impacting the overall performance. From the above description, it is apparent that the photoresist pattern replicates the photomask pattern, while the photomask pattern is drawn by computer-aided design software. Thus, one can utilize computer-aided design software to design the shape and size of the final pattern freely, which is especially useful in designing MBs where intricate patterns are required.

## Specific Applications of Photolithography in MBs

**Fig. 2:**
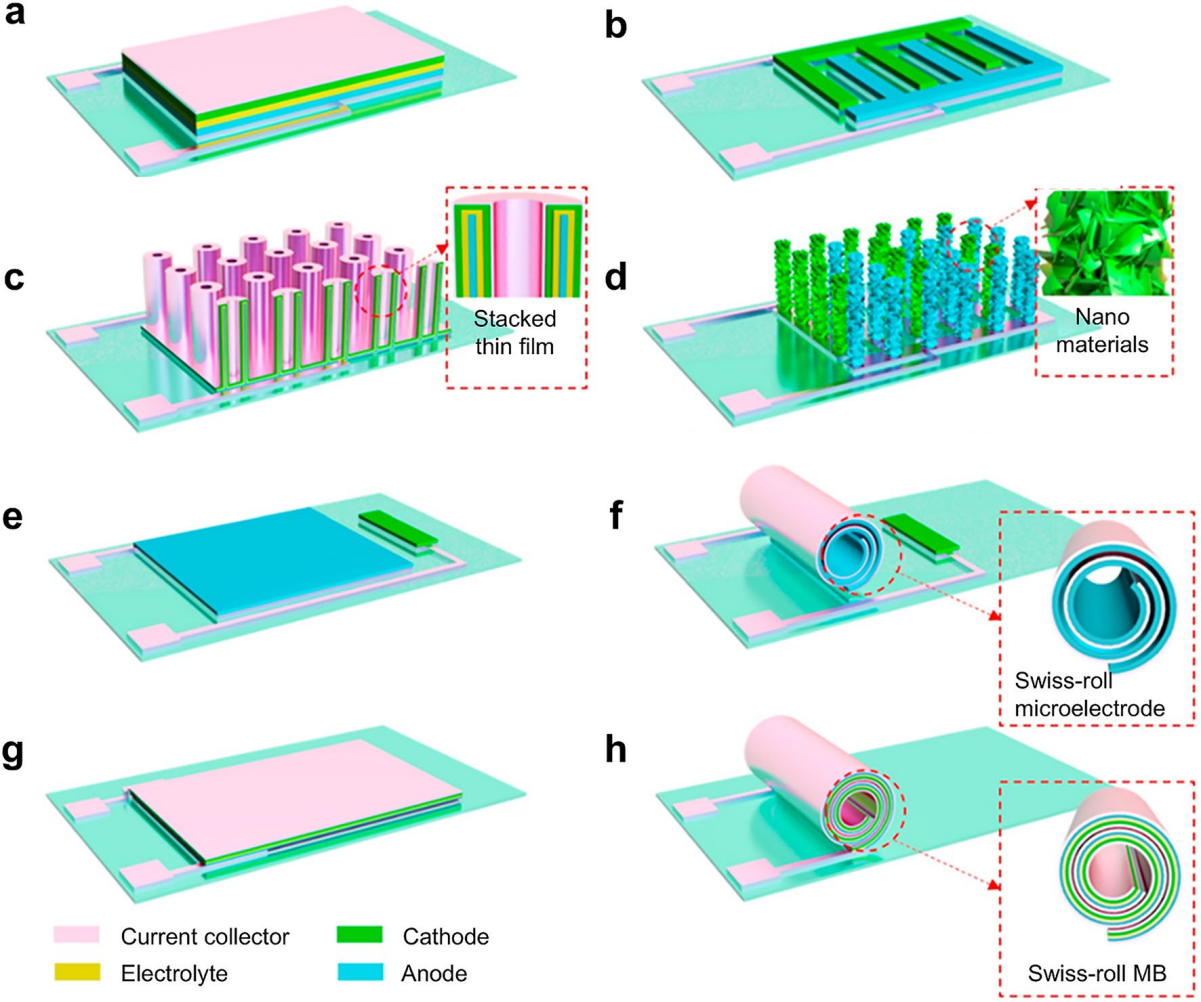
Schematic illustration of different configurations of MBs. **a** Stacked geometry of MB. **b** In-plane geometry of MB. **c** 3D stacked geometry of MB. **d** 3D in-plane geometry of MB. In-plane geometry of MB **e** before and **f** after being constructed into Swiss-roll configuration. Stacked geometry of MB **g** before and **h** after being constructed into Swiss-roll configuration. **a**–**h** Reproduced with permission from Ref. [12]. Copyright 2021, Elsevier

So far, there are two common MB structures of stacked geometry and in-plane geometry (Fig. 2a, b). In the stacked geometry, the current collector (on the cathode side), cathode, electrolyte, anode, and current collector (on the anode side) are systematically layered, thereby forming the comprehensive battery architecture (Fig. 2a). For the in-plane geometry, the cathode and anode (both in contact with their respective current collectors) are located on the same plane with a gap between them to prevent short circuits. The electrolyte permeates the electrodes and fills the gap between the cathode and the anode. In practical application, the cathode and anode of the in-plane geometry are generally designed as interdigital shapes to increase the contact area between the electrode and electrolyte (Fig. 2b).

In recent years, many studies on MBs have focused on improving the areal energy density, which requires MBs to load more electrode materials within a given unit area. The most intuitive and direct method is to increase the thickness of the electrode materials. However, merely increasing the thickness of electrode materials will prolong the transmission path of electrons and ions, consequently hindering the charge transfer reaction and deteriorating the rate performance of MBs. At the same time, during the charging and discharging process, the volume of active materials changes due to the intercalation and deintercalation of ions. Thickening electrode materials will make the effect of volume change more significant, resulting in cracks or even collapse of the electrode structure, thereby truncating the cycle life of MBs [12].

To counter these issues, some researchers proposed a 3D configuration strategy. Leveraging micro-machining technology, these strategies meticulously design and construct 3D architectures, such as holes, pillars, and trenches at micro or even nanoscales (Fig. 2c, d) to increase the load of electrode materials without compromising the power density and cycle life, and ultimately boosting the energy density of MBs [1, 4, 11, 12]. In addition, researchers also used the microscopic self-assembly method to build the MB into a micro-Swiss-roll structure (Fig. 2e–h), thereby shrinking the footprint of the MB without reducing the load of the electrode materials to achieve an increase in areal energy density [12]. Photolithography technology plays a pivotal role in the fabrication of MB and 3D MBs, leveraging its high precision, unparalleled efficiency, scalability, and flexibility to provide crucial support for enhancing battery performance.

**Fig. 3:**
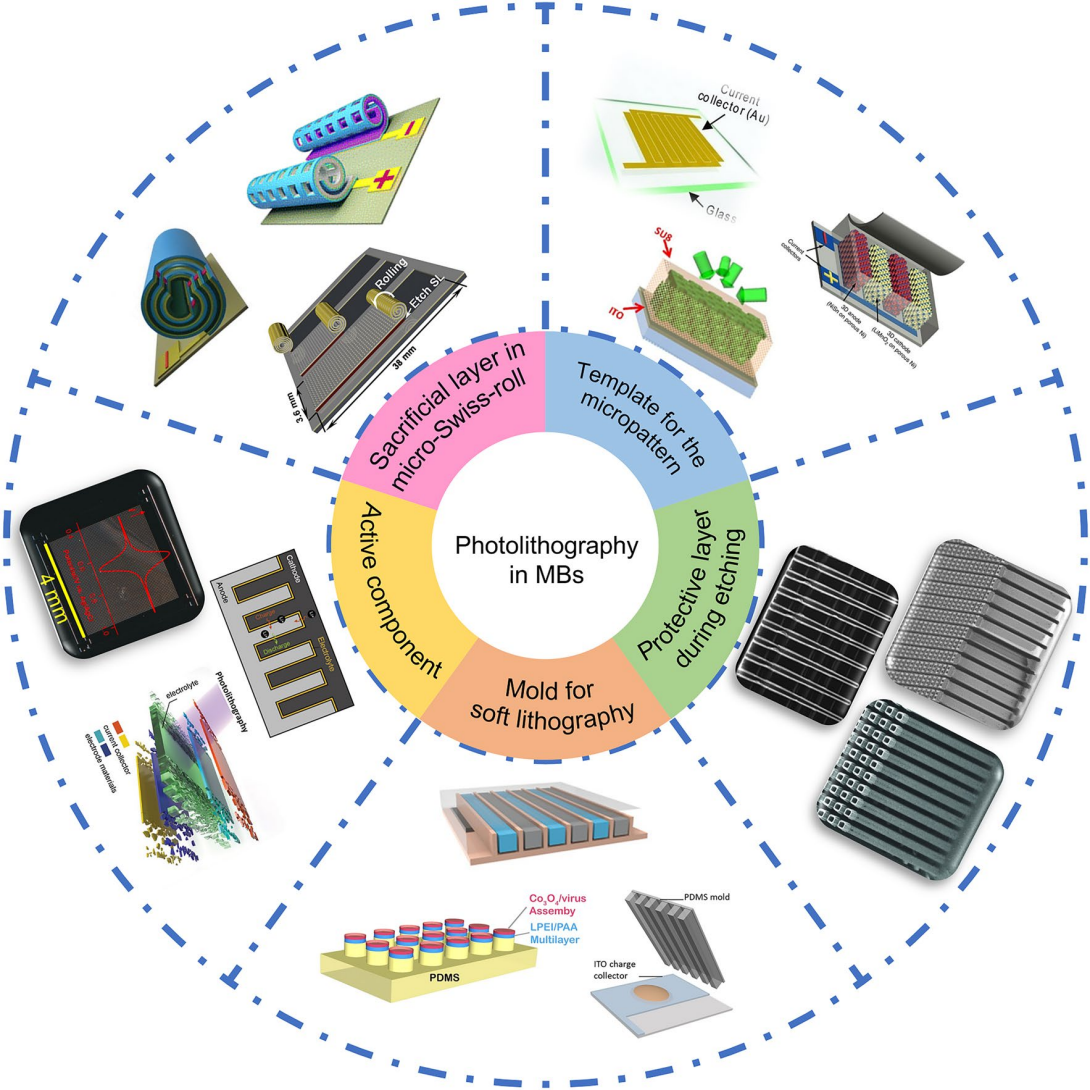
Applications of photolithography in MBs, which include the template for the micropattern, protective layer during etching, mold for soft lithography, active component, and sacrificial layer in micro-Swiss-roll. Reproduced with permission from Ref. [51]. Copyright 2013, John Wiley and Sons. Reproduced with permission from Ref. [71]. Copyright 2013, Springer Nature. Reproduced with permission from Ref. [58]. Copyright 2015, National Academy of Sciences, U.S.A. Reproduced with permission from Ref. [72]. Copyright 2010, Royal Society of Chemistry. Reproduced with permission from Ref. [73]. Copyright 2014, John Wiley and Sons. Reproduced with permission from Ref. [74]. Copyright 2017, John Wiley and Sons. Reproduced with permission from Ref. [75]. Copyright 2020, John Wiley and Sons. Reproduced with permission from Ref. [76]. Copyright 2008, National Academy of Sciences, U.S.A. Reproduced with permission from Ref. [59]. Copyright 2018, Elsevier. Reproduced with permission from Ref. [77]. Copyright 2018, Elsevier. Reproduced with permission from Ref. [78]. Copyright 2019, American Chemical Society. Reproduced with permission from Ref. [65]. Copyright 2024, John Wiley and Sons. Reproduced with permission from Ref. [18]. Copyright 2022, John Wiley and Sons. Reproduced with permission from Ref. [64]. Copyright 2023, Royal Society of Chemistry. Reproduced with permission from Ref. [63]. Copyright 2021, Elsevier

In the subsequent sections, we will delve into the application of photolithography in MBs, including the construction of 2D and 3D MB structures. Photolithography allows for the precise formation of 2D in-plane geometry, including interdigital designs for enhanced electrode–electrolyte contact area. For 3D geometry, photolithography can be combined with other techniques, such as etching or electrodeposition, to create complex structures like holes, pillars, and trenches. Moreover, photolithography has found novel applications beyond structural construction. For instance, it has emerged as a versatile tool for creating functional layers in MBs, such as the protective layer, sacrificial layer, and selective ion-permeable membranes (Fig. 3). In the following discussions, we aim to provide a comprehensive overview of these diverse applications, elucidating the versatility and potential of photolithography in the realm of MBs.

### Template for the Micropattern Processing of MBs

As the above description shows, photolithography is a powerful technology for precisely fabricating micropatterns with very high resolution. Thus, the most widespread application of photolithography in MBs is to construct the template to define the intricate pattern of MB components, including the current collectors and the electrodes, especially the interdigital patterns for the in-plane geometry.

**Fig. 4:**
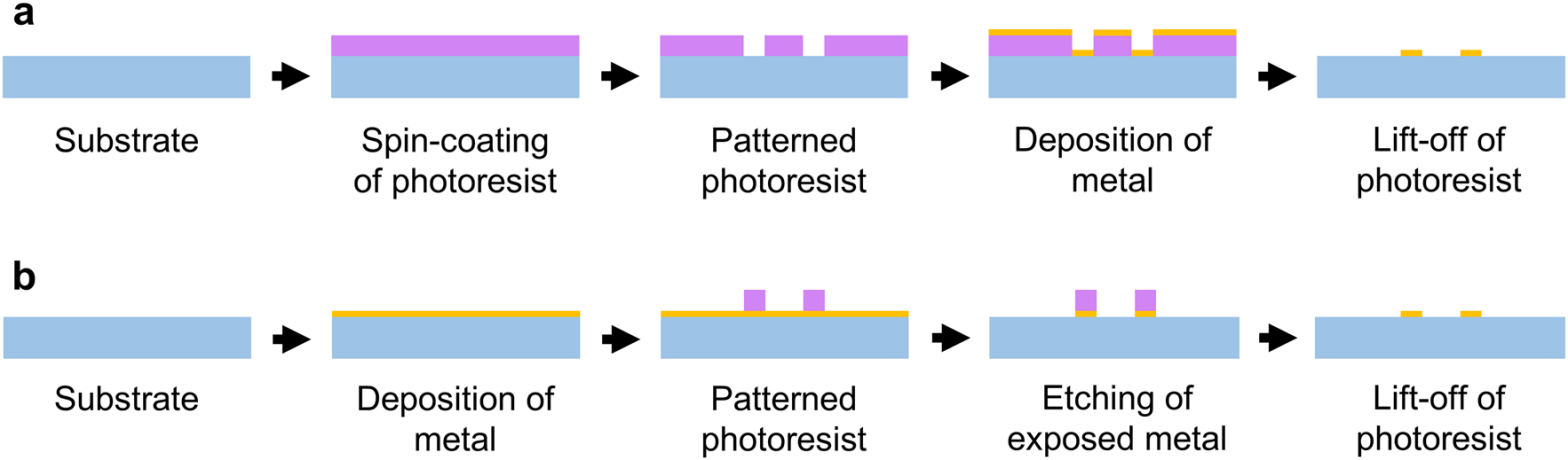
Schematic illustration of two photolithography methods of fabricating interdigital current collectors. **a** Patterned photoresist is prepared on the substrate first, and then the metal is deposited. Following the removal of the photoresist, the interdigital current collector is obtained. **b** Metal is deposited on the substrate first, and then, the patterned photoresist is prepared. Following the etching of exposed metal and the removal of the photoresist, the interdigital current collector is obtained

Specifically, after photolithography, a metal film is deposited on the photoresist-patterned surface using magnetron sputtering or thermal evaporation techniques, followed by a lift-off process. The lift-off process soaks the substrate in a solvent, and the photoresist can dissolve in the solvent, causing the metal to fall off its surface and leaving the final patterned current collector (Fig. 4a). Another key strategy to fabricate the micropatterned current collector is to deposit the metal on the substrate before photolithography. After photolithography, the patterned photoresist will cover the metal beneath it and protect it during etching, while the uncovered metal will be etched. After removing the remaining photoresist, the interdigital current collector can also be obtained (Fig. 4b).

**Fig. 5:**
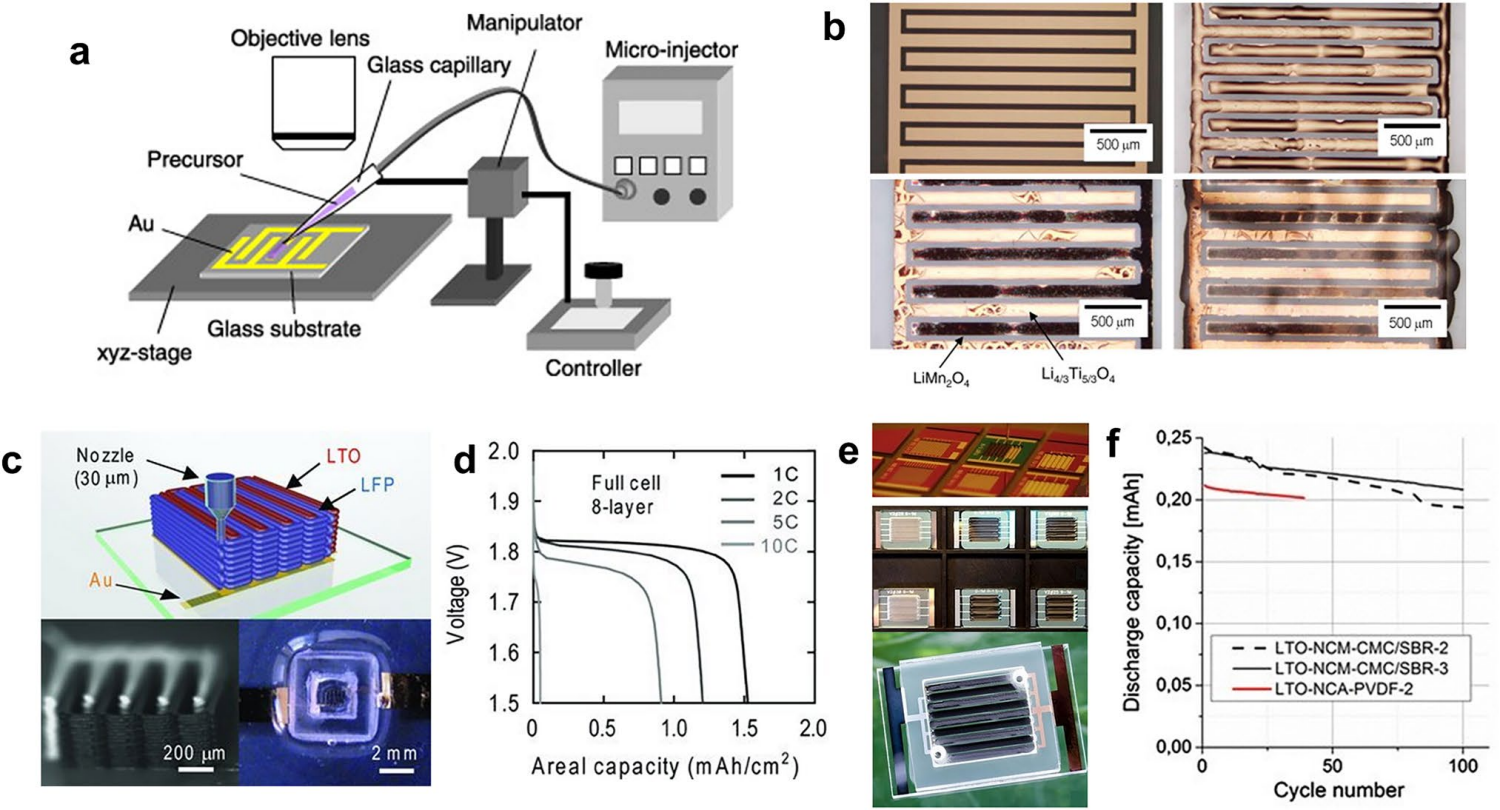
Application of micro-injection and 3D printing technology for loading electrode materials on the current collectors. **a** Schematic illustration of the micro-injection system for fabricating interdigital electrodes. Reproduced with permission from Ref. [79]. Copyright 2007, Elsevier. **b** Optical microscope images of the interdigital current collector fabricated by photolithography, the precursor sol injected onto the current collector, the prepared electrode materials, and the MB after casting the electrolyte film. Reproduced with permission from Ref. [80]. Copyright 2007, Elsevier. **c** Schematic illustration of the 3D MBs fabricated by 3D printing and optical images of 3D interdigital architecture before and after packaging. **d** Discharge curves of the 3D MBs at different current densities. **c**, **d** Reproduced with permission from Ref. [51]. Copyright 2013, John Wiley and Sons. **e** Optical image of dispensing the electrode into cavities and MBs with interdigitated electrodes packaged with a glass lid. **f** Cycling performance of the MBs dispensed with different electrode materials at the current density of 0.5 C. **e**, **f** Reproduced with permission from Ref. [84]. Copyright 2022, Springer Nature

After the preparation of the current collector, the electrode materials need to be loaded onto it to complete the fabrication of MBs. The most intuitive way is to place the electrode materials directly on the current collectors using micro-injection or 3D printing technology. For example, Kanamura and coworkers used a glass capillary and a micro-injection system to inject the electrode precursor sols onto the interdigital current collectors fabricated by photolithography (Fig. 5a) [79, 80]. With the help of hydrophobic and hydrophilic pretreatment of the substrate and current collectors, the sols could be precisely loaded. After the precursor sol was dried into a gel and calcinated, the microarray electrodes of the LiMn_2_O_4_ cathode and Li_4/3_Ti_5/3_O_4_ (LTO) anode were synthesized on the current collectors (Fig. 5b). The MB could deliver a specific capacity of around 4.5 μAh cm^−2^ and an energy density of 11 μWh cm^−2^, which are relatively low. The main reason for its low areal capacity may be that the electrode material precursor content in the sol is too small, resulting in a low loading of the electrode material. In contrast, the content of electrode material in 3D printing ink is relatively large, and the electrode ink can be processed by its unique fluid rheological properties so that the capacity of the battery can be increased and the electrode material can be precisely loaded [81–83]. For instance, Lewis et al. fabricated the interdigital current collectors by photolithography and printed the LiFePO_4_ (LFP) cathode and the LTO anode on the current collectors, forming a 3D interdigitated MB architecture (Fig. 5c) [51]. With the advantage of multi-layer printing of electrode materials, the 3D MBs could achieve a high areal capacity of 1.5 mAh cm^−2^ (Fig. 5d) and a high energy density of 9.7 J cm^−2^ (2.7 mWh cm^−2^). Besides, Hahn et al. fabricated substrates with cavities by direct laser ablation and constructed the current collectors by photolithography [84]. The electrode materials, such as LTO or graphite as anode and LiNi_1/3_Co_1/3_Mn_1/3_O_2_ (NCM) or LiNi_0.8_Co_0.15_Al_0.05_O_2_ (NCA) as cathode, were then filled in the cavities by micro-dispensing. Followed by electrolyte filling and packaging, the wafer-scale MB fabrication was achieved (Fig. 5e). Due to the great depth of the cavities (200 μm), the electrode materials could be stably loaded with a large amount, and the fabricated MB could deliver an areal capacity of 0.625 mAh cm^−2^. The dedicated packaging technique also enabled the MBs to have capacity retention of 89% after 100 cycles (Fig. 5f).

**Fig. 6:**
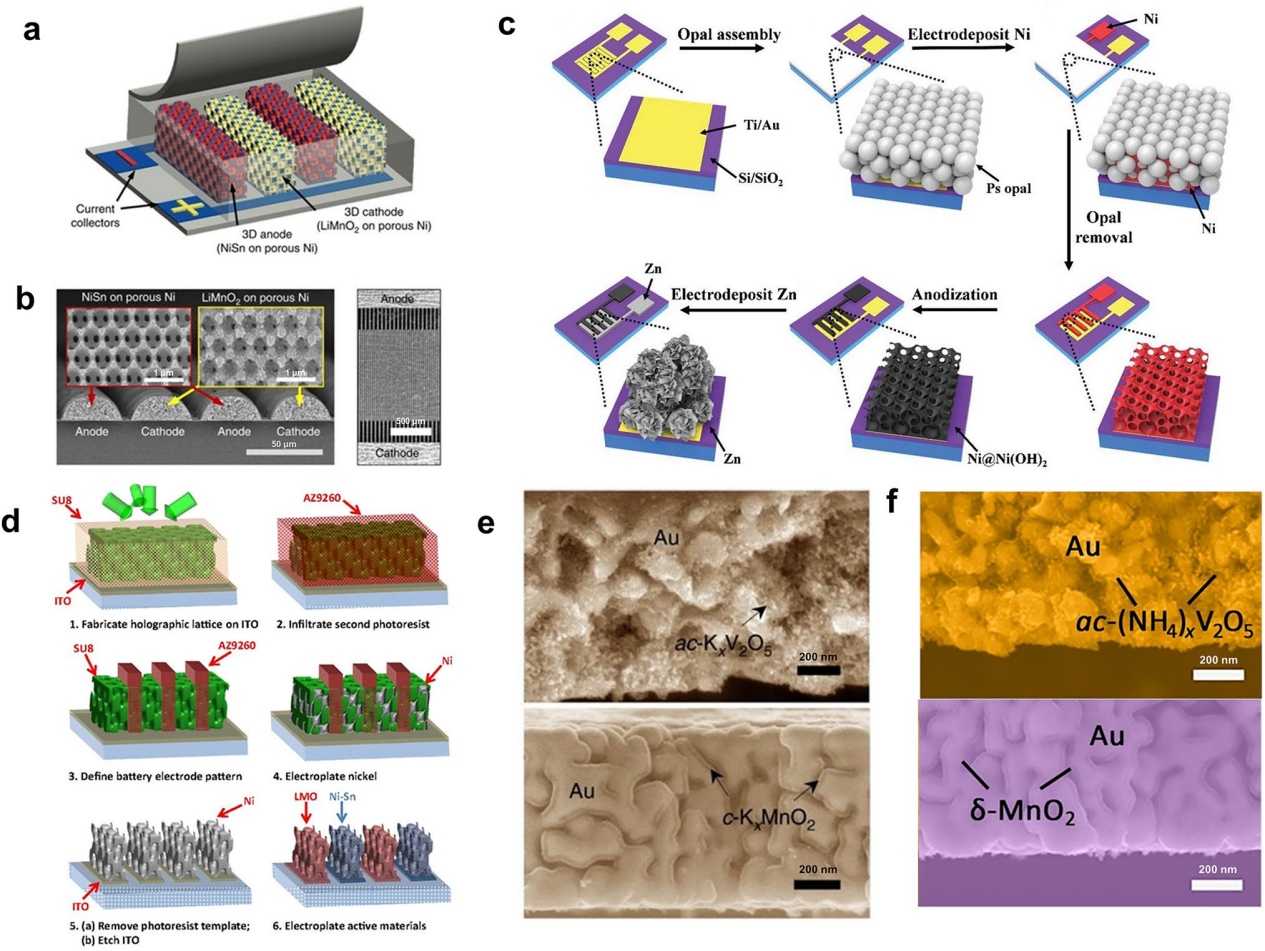
Application of electrodeposition technology for loading electrode materials on the 3D porous current collectors. **a** Schematic illustration of the 3D porous MBs fabricated by PS nanospheres. **b** Scanning electron microscopy (SEM) images of the 3D porous interdigitated electrodes fabricated by PS nanospheres. **a**, **b** Reproduced with permission from Ref. [71]. Copyright 2013, Springer Nature. **c** Schematic illustration of the fabrication process of hierarchically ordered porous Ni–Zn MBs. Reproduced with permission from Ref. [88]. Copyright 2019, John Wiley and Sons. **d** Schematic illustration of the MB fabricated by holographic patterning. Reproduced with permission from Ref. [58]. Copyright 2015, National Academy of Sciences, U.S.A. **e** Cross-sectional SEM images of the K_x_V_2_O_5_ and K_x_MnO_2_ supported by nanoporous Au current collectors. Reproduced with permission from Ref. [91]. Copyright 2019, Springer Nature. **f** Cross-sectional SEM images of 3D nanoporous Au supported (NH_4_)_x_V_2_O_5_ and *δ*-MnO_2_. Reproduced with permission from Ref. [92]. Copyright 2023, Elsevier

Another method of loading electrode materials onto the current collectors is electrodeposition. Since the active material is attracted by the potential difference generated at the current collector, the cathode and anode materials can be precisely deposited onto their respective current collectors, thus avoiding the occurrence of short circuits [85]. The current collectors are usually made into a 3D structure to enhance the capacity and rate performance of the MBs, which can increase the mass loading of the electrode materials and improve the transport of ions and electrons while maintaining a small footprint area [1, 2, 4, 12, 20, 22, 86]. For example, Braun and coworkers made the 3D bicontinuous porous current collectors by self-assembling polystyrene (PS) nanospheres onto the 2D interdigital current collectors, electrodepositing of Ni, and etching of PS nanospheres [71]. Following electrodepositing the LiMnO_2_ cathode and NiSn anode, the MBs with 3D bicontinuous nanoporous electrodes were prepared (Fig. 6a, b). This work illustrates how photolithography can be combined with other techniques, such as self-assembly, electrodeposition, and etching, to create complex MB structures. Owing to the 3D porous structure of the electrode, the MB could even work at a very high current density of 1000 C, and the corresponding power density was 7360 μW cm^−2^ μm^−1^. Using a similar method, they fabricated the primary MBs with the 3D porous manganese oxide cathode and bulky Li anode [87]. The primary MB could achieve 45.5 μWh cm^−2^ μm^−1^ energy and 5300 μW cm^−2^ μm^−1^ peak power density. Further, Mai et al. used a similar method to fabricate the Ni − Zn MBs with hierarchically ordered porous Ni@Ni(OH)_2_ cathode based on a 3D current collector and bulky Zn anode (Fig. 6c) [88]. Also, benefiting from the interconnected ordered electrolyte-filled macropore–mesopore network, the MB delivered an impressive energy density of 0.26 mWh cm^−2^ and a power density of 33.8 mW cm^−2^.

Besides using the PS nanosphere as the template, Braun’s group also developed a holographic patterning method to prepare the 3D current collector and porous electrode [58]. With the help of four coherent laser beams distributed in an umbrella geometry, the periodic light intensity arrangement in 3D space is realized by the interference between the four coherent laser beams [89]. From the mechanism of photolithography described above, it is foreseeable that the photoresist exposed to such light will be transformed into a 3D porous structure after the development process. After the traditional 2D photolithography process to define the interdigital pattern of the current collector, the 3D porous electrode architecture can be obtained by electrodeposition of Ni current collector and removing the 3D porous photoresist. Following the electrodeposition of the LiMnO_2_ cathode and NiSn anode, the holographic-patterned MB was obtained (Fig. 6d). This method can avoid the structural irregularity that may occur during the self-assembly of PS nanospheres, facilitating the structural integrity of the whole electrode. Additionally, the size and arrangement of the porous structure can be conveniently controlled by adjusting the parameters of the light source, providing the freedom to alter the electrode structure. The MB fabricated by holographic patterning could also discharge at the current density of 1000 C, and the cycling performance was also improved (77% after 200 cycles) by carefully matching the electrode capacities and precycling the anode to form a solid electrolyte interphase layer.

The 3D porous electrode can also be fabricated by dealloying, which contains the construction of the interdigital alloy current collector and selective dissolution of the more electrochemically active element, leaving behind a porous structure composed of the more inert alloy constituent [90]. For instance, Jiang and colleagues reported a porous Au collector by dissolving Ag from Ag_75_Au_25_ (at%) alloy in concentrated HNO_3_ [91]. After the electrodeposition of K_x_V_2_O_5_·nH_2_O anode and K_x_MnO_2_·nH_2_O cathode (Fig. 6e), the obtained potassium ion MB could reach a maximum energy density of 103 mWh cm^−3^ and power density of 600 W cm^−3^. In the same way, Li et al. electrodeposited the (NH_4_)_x_V_2_O_5_ anode and* δ*-MnO_2_ cathode onto the nanoporous Au current collectors fabricated by dealloying and obtained an ammonium ion MB (Fig. 6f) [92]. The MB offered an energy density of 0.126 Wh cm^−3^ and maintained 93.3% of its initial capacity after 10,000 cycles.

### Protective Layer for the Etching Process

Besides the methods described above, RIE and deep reactive ion etching (DRIE) of Si, widely utilized in the semiconductor industry [93], are also developed in MB manufacturing to make the microelectrodes into a 3D structure. The Si substrates are usually etched into micropillar arrays or micro hole arrays, which are the basis of the 3D structure of the electrodes. The size, shape, and pitch of these pillars or holes of the final etched pattern are determined by photolithography. Specifically, the photoresist can protect the Si beneath it, while the exposed Si will be etched away, resulting in a 3D structure corresponding to the pattern defined by the photolithography. The electrode materials and the electrolyte will then be deposited using various conformal deposition methods to complete the fabrication of MBs. The construction of the 3D electrode structure can make full use of the limited space, increase the mass loading of the electrode material per unit projected area, and then enhance the areal capacity and energy density of MBs.

**Fig. 7:**
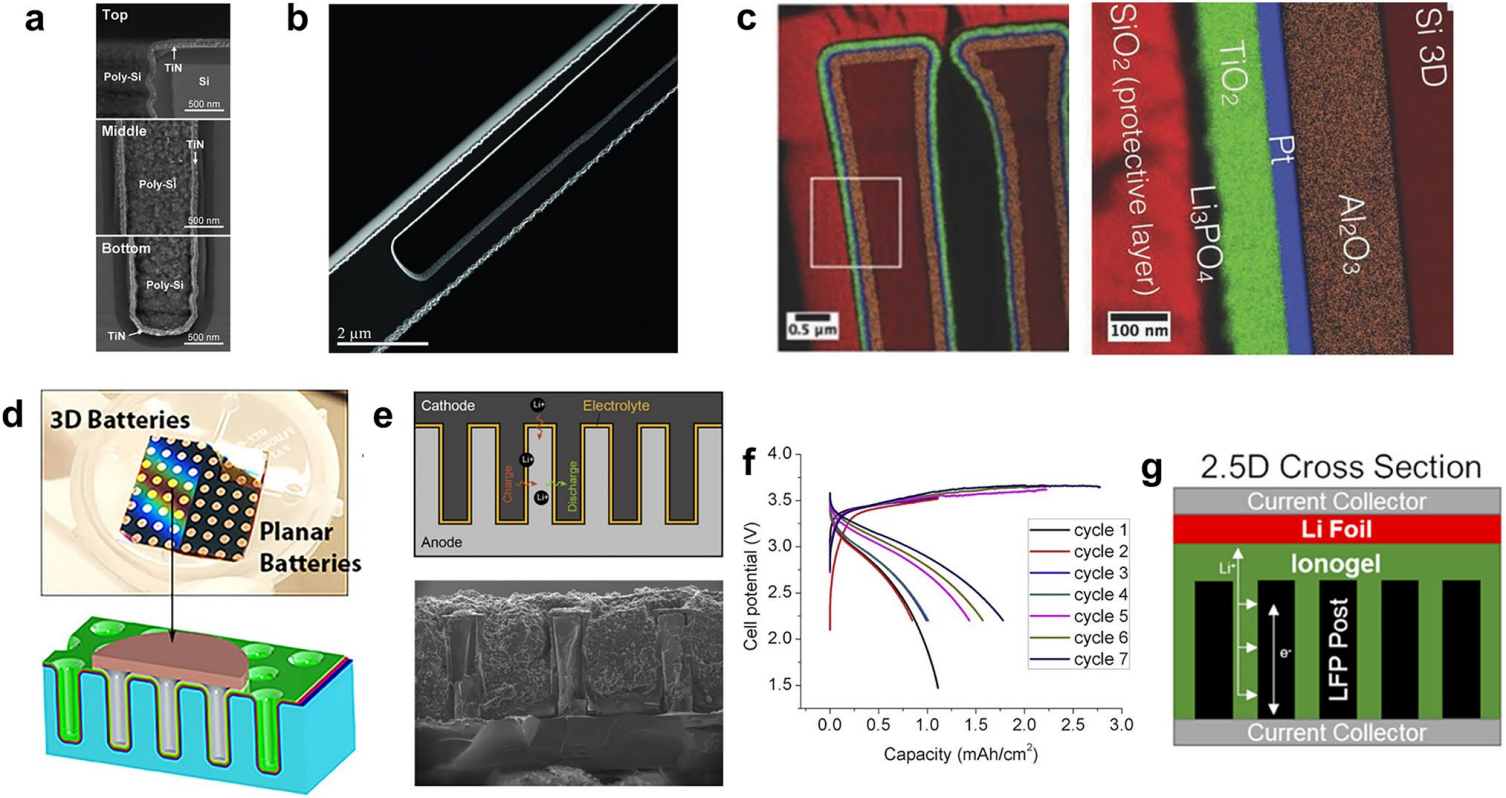
Application of Si etching technology for fabricating 3D electrode architecture. **a** High-resolution SEM images showing the Si substrate covered by conformal TiN and poly-Si thin films. Reproduced with permission from Ref. [72]. Copyright 2010, Royal Society of Chemistry. **b** Scanning transmission electron microscopy (STEM) images illustrating the conformal deposition of Pt/TiO_2_ on the Si microtube. Reproduced with permission from Ref. [73]. Copyright 2014, John Wiley and Sons. **c** Energy dispersive X-ray spectroscopy STEM (EDX-STEM) mapping of coated double microtubes exhibiting the stacked layers of Si-3D/Al_2_O_3_/Pt/TiO_2_/Li_3_PO_4_/SiO_2_-protective. Reproduced with permission from Ref. [74]. Copyright 2017, John Wiley and Sons. **d** Optical image and schematic illustration of SnN_x_||LiV_2_O_5_ 3D full-cells chip. Reproduced with permission from Ref. [68]. Copyright 2018, American Chemical Society. **e** Schematic illustration and SEM image of Si||NCA full 3D battery. **f** Galvanostatic charge and discharge (GCD) profiles of Si||NCA full 3D battery at different cycles. **e**, **f** Reproduced with permission from Ref. [77]. Copyright 2018, Elsevier. **g** Schematic illustration of the Li||LFP 2.5D MB. Reproduced with permission from Ref. [96]. Copyright 2020, American Chemical Society

Representatively, Golodnitsky and coworkers utilized photolithography and DRIE to fabricate the through-hole arrays on Si substrate [94]. Followed by the electroless plating of the Ni current collector and electrodeposition of MoS_2_, a cathode with a 3D microstructure was obtained. The 3D MoS_2_ cathode could deliver a high capacity of 1 mAh cm^−2^ owing to the increase of the geometrical area, which was about 20 times that of a planar cathode. Moreover, Notten et al. used photolithography and RIE to produce the Si substrate with pores and trenches [72]. Then, the TiN current collector and the polycrystalline Si (poly-Si) electrode were conformally deposited by atomic layer deposition (ALD) and low-pressure chemical vapor deposition, respectively (Fig. 7a). The 3D poly-Si achieved an initial capacity of 250 μAh cm^−2^. However, it suffered from severe capacity fading (less than 20% after 100 cycles). This is attributed to the continual growth of the solid electrolyte interphase, which would block the pores and trenches, limit the kinetics of the electrochemical reaction kinetics, and reduce the storage capacity. Similarly, Lethien’s group created the 3D Si microtube scaffold and deposited the Pt current collector and TiO_2_ electrode by the ALD process (Fig. 7b) [73]. The results showed that with the help of the 3D scaffold, a 120 nm thick TiO_2_ half-cell could provide a capacity of 0.2 mAh cm^−2^. On this basis, they integrated the solid electrolyte into the 3D scaffold in a later study. After the fabrication of the 3D scaffold and the conformal deposition of the Al_2_O_3_ insulating layer, Pt current collector, and TiO_2_ electrode, the Li_3_PO_4_ solid electrolyte was also deposited by ALD (Fig. 7c) [74]. The deposited pinhole-free Li_3_PO_4_ solid electrolyte had a high ionic conductivity of 6.2 × 10^−7^ S cm^−1^ and a low thickness layer of 10 nm, enabling the TiO_2_ electrode to obtain a capacity of 0.37 mAh cm^−2^. In addition, Pearse and coworkers also fabricated the 3D Si substrate by photolithography and DRIE, and the Ru current collector (cathode side), LiV_2_O_5_ cathode, LiPON solid-state electrolyte, SnN_x_ anode, TiN current collector (anode side) were all deposited by the ALD process [68]. Afterward, Cu was deposited as the probe contact and etching mask by electron beam deposition, and the electrical isolation of adjacent MBs was realized by etching the exposed SnN_x_ anode and TiN current collector. Thus, the scalable fabrication of the micro full-cells was successfully achieved (Fig. 7d). The micro full-cell could deliver a capacity of 29 μAh cm^−2^, 9.3 times higher than the planar micro full-cell. In addition to holes or trenches fabricated in the work mentioned above, pillars and posts can also be constructed to enhance the performance of MBs. For example, Notten’s group used photolithography and DRIE to fabricate the Si nano-pillar arrays and deposited the TiN current collector and TiO_2_ electrode by ALD and metal–organic chemical vapor deposition [95]. The 3D TiO_2_ electrode could deliver a capacity of about 210 μAh cm^−2^ μm^−1^ at the current density of 4 μA cm^−2^, and a capacity of around 66 μAh cm^−2^ μm^−1^ could be maintained at the elevated current density of 80 μA cm^−2^. After 380 cycles, no capacity attenuation was found.

It should be noted that the protective layer during Si etching can also be SiO_2_ other than the photoresist. Specifically, SiO_2_ is deposited first on the Si substrate surface. After the photolithography process, the photoresist-uncovered SiO_2_ can be etched first, exposing the Si substrate beneath it. Followed by removing the photoresist and the DRIE process (SiO_2_ is the protective layer at this time), the 3D structure can also be obtained. After removing the remaining SiO_2_ and the etched byproducts, the whole process is finished. Typically, Dunn’s group used this method to construct the 3D Si post anode, and a photopatternable polymer electrolyte (more on this in the following section) and NCA cathode were elaborately incorporated, forming the final MBs (Fig. 7e) [77]. Owing to the high mass loading of the electrode and high working potential differences between the NCA cathode and Si anode, the MB could deliver a capacity of 1.8 mAh cm^−2^ (Fig. 7f) and a very high energy density of 5.2 mWh cm^−2^. Besides, the same group created a “2.5D MB” by mixing the 3D LFP post cathode and 2D planar Li anode [96]. The 3D LFP post was fabricated by high-pressure injection and vacuum infiltration into a mold made of a 3D Si hole array and etching of the Si mold. After filling the ion-gel electrolyte and pressing the planar Li electrode, the 2.5D MB was constructed (Fig. 7g). This work greatly simplified the preparation process of the MBs without compromising the electrochemical performance. Remarkably, a high capacity (1.4 mAh cm^−2^) and a high energy density (5 mWh cm^−2^) could still be reached.

### Mold for Soft Lithography

**Fig. 8:**
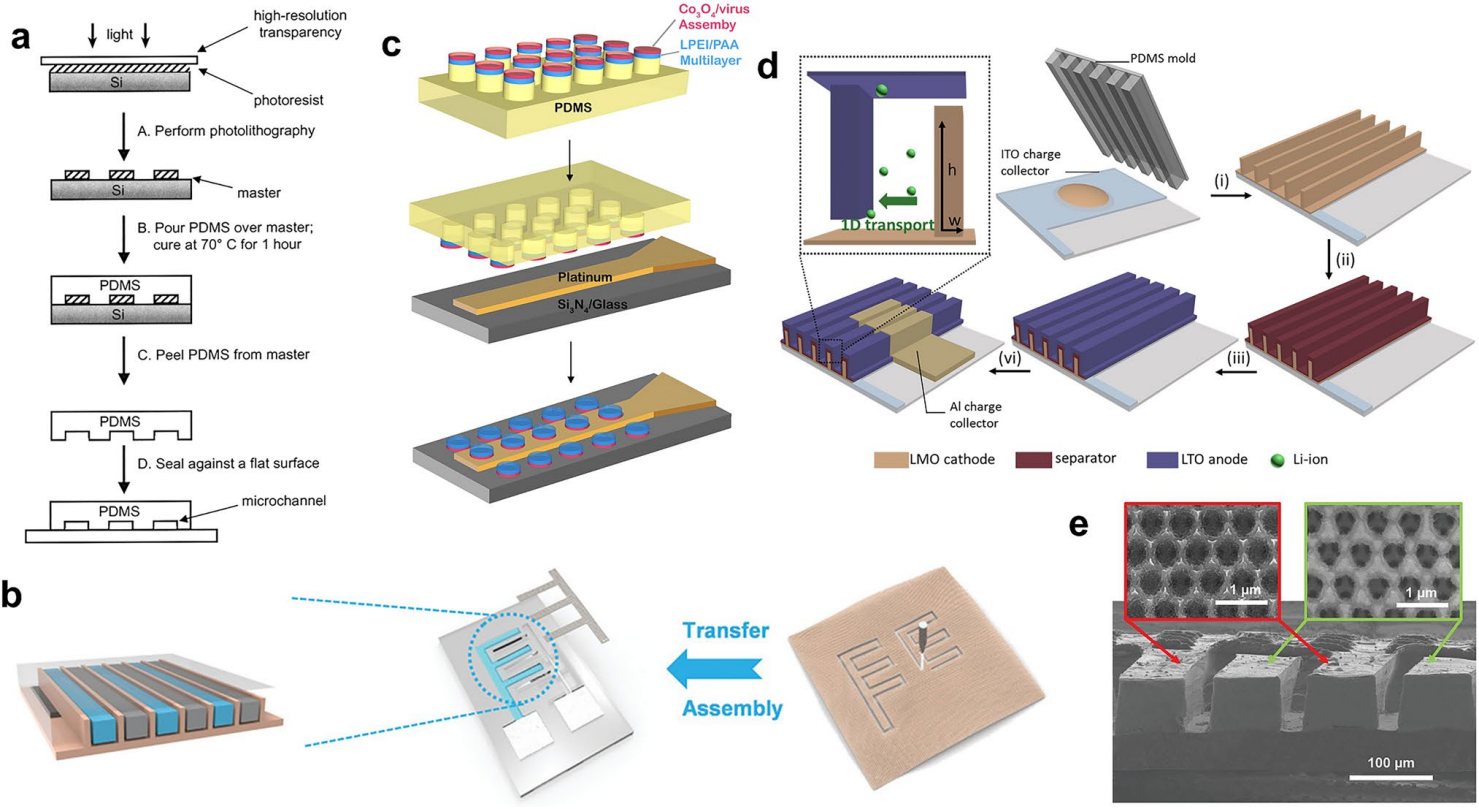
Application of soft lithography in MBs. **a** Schematic illustration of the fabrication steps of a microfluidics chip made by PDMS. Reproduced with permission from Ref. [100]. Copyright 2002, American Chemical Society. **b** Schematic illustration of transferring and assembling the Co–Zn MB onto PDMS and the side-view of the fabricated MB. Reproduced with permission from Ref. [75]. Copyright 2020, John Wiley and Sons. **c** Schematic illustration of the procedure for constructing virus-based MB electrodes. Reproduced with permission from Ref. [76]. Copyright 2008, National Academy of Sciences, U.S.A. **d** Schematic illustration of 1D transport in the interdigitated microelectrode array and LTO||LiMn_2_O_4_ MB fabrication steps. Reproduced with permission from Ref. [59]. Copyright 2018, Elsevier. **e** Cross-section SEM image of the interdigitated V_2_O_5_ and Li electrodes. Reproduced with permission from Ref. [60]. Copyright 2021, John Wiley and Sons

Another function of photolithography is that it can serve a pivotal role in producing molds for soft lithography. Soft lithography is a method that uses elastomeric stamps, molds, and conformable photomasks for patterning 2D and 3D structures [97]. The process commences with fabricating a hard mold bearing a specific pattern. Patterned photoresist can be directly employed as a hard mold, or a Si-based hard mold can be fabricated by applying photolithography and etching techniques. Subsequently, a liquid polymer or polymer monomer will be cast onto the hard mold and undergo a curing process, during which the pattern is transferred from the hard mold to the polymer, resulting in a soft mold. The soft mold can be used for micro imprinting or as a 3D substrate. Since Whitesides’s group developed the preparation process of poly(dimethylsiloxane) (PDMS) soft mold and successfully applied it to microfluidic chips (Fig. 8a) [98–100], PDMS has become the most popular soft mold. For the processing of PDMS, two prepolymers, hydrosiloxane (containing –Si–H group) and vinvlsiloxane (containing –Si–CH=CH_2_ group) [101], will be thoroughly mixed and poured onto the hard mold and then heated. During heating, cross-linking will happen between –Si–H and –Si–CH=CH_2_ to form –Si–CH_2_–CH_2_–Si–, and the cured PDMS is obtained [102]. After cooling to room temperature, the PDMS will be peeled off from the hard mold, yielding a PDMS soft mold with a specific pattern. For instance, Mai et al. employed direct laser engraving to fabricate the interdigital textile-based Co(OH)_2_@NiCo-layered double hydroxide cathode and Zn anode of the Co−Zn MB, and the interdigital cathode and anode were put into a PDMS interdigital groove made by photoresist hard mold (Fig. 8b) [75]. Due to the inherent flexibility of PDMS, it could be used as a wearable substrate to accommodate electrode materials and electrolytes. The wearable MB could deliver an energy density of 0.17 mWh cm^−2^ and a power density of 14.4 mW cm^−2^, and the capacity retention is 71% after 800 cycles. In addition to serving as a flexible substrate, the soft mold can be used as a stamp to load materials and create 3D structures. Further, Chiang et al. used the photoresist hard mold to fabricate the PDMS microcylindrical arrays [76]. Then, the linear-polyethylenimine (LPEI)/polyacrylic acid (PAA) solid electrolyte was deposited on the PDMS microcylinder by alternating electrostatic layer-by-layer assembly. After that, the modified M13 virus solution was dropped on LPEI/PAA, and the Co_3_O_4_ nanowires electrode was self-assembled. Finally, the micro-cylinder arrays were stamped onto the Pt microband current collector, forming the final microelectrodes (Fig. 8c). The microelectrode array showed a discharge capacity ranging from 375 to 460 nAh at a low discharge current (26 nA). When the discharge current was elevated to 255 nA, the capacity was 100–150 nAh. Watkins and coworkers also used the PDMS to create the 3D structure of the microelectrodes [59]. The LiMn_2_O_4_ cathode ink was first spin-coated onto the ITO-coated glass substrates and then stamped with a PDMS fabricated by hard mold to build a 3D cathode. After casting the gel electrolyte, spin-coating the LTO anode ink, and thermally evaporating the Al current collector, the 3D MB was obtained (Fig. 8d). Compared to the nonpatterned electrode, the 3D structure improved the rate performance of the MB dramatically. When the current density is 300 C, about 40% of the capacity at 5 C could be retained, resulting in a maximum power density of 855.5 μW cm^−2^ μm^−1^. In addition to PDMS, other soft molds can also be used to prepare the 3D structure of the electrode. For example, Bruan et al. used Si hard mold to fabricate the poly(methyl methacrylate) (PMMA) interdigital soft mold, and the 3D porous Ni current collector was prepared by the PS nanospheres-assisted electrodeposition described in the previous section [60]. Due to the space restriction of the deep PMMA trench, the Ni current collector could be electrodeposited in the vertical direction, which took full advantage of the vertical dimension and increased the mass loading of electrode materials in the unit footprint (Fig. 8e). After transferring the Ni current collector onto the polyimide substrate, incorporating the V_2_O_5_ cathode, Li anode, and electrolyte, and packaging with UV-curable adhesive, the fabrication of the 3D MB was finished. The packaged MB could achieve a high energy/power density of 1.242 J cm^−2^ (0.345 mWh cm^−2^)/75.5 mW cm^−2^. After 200 cycles in the air, the packaged MB retained 75% of the initial discharge capacity.

### Active Component of MBs

**Fig. 9:**
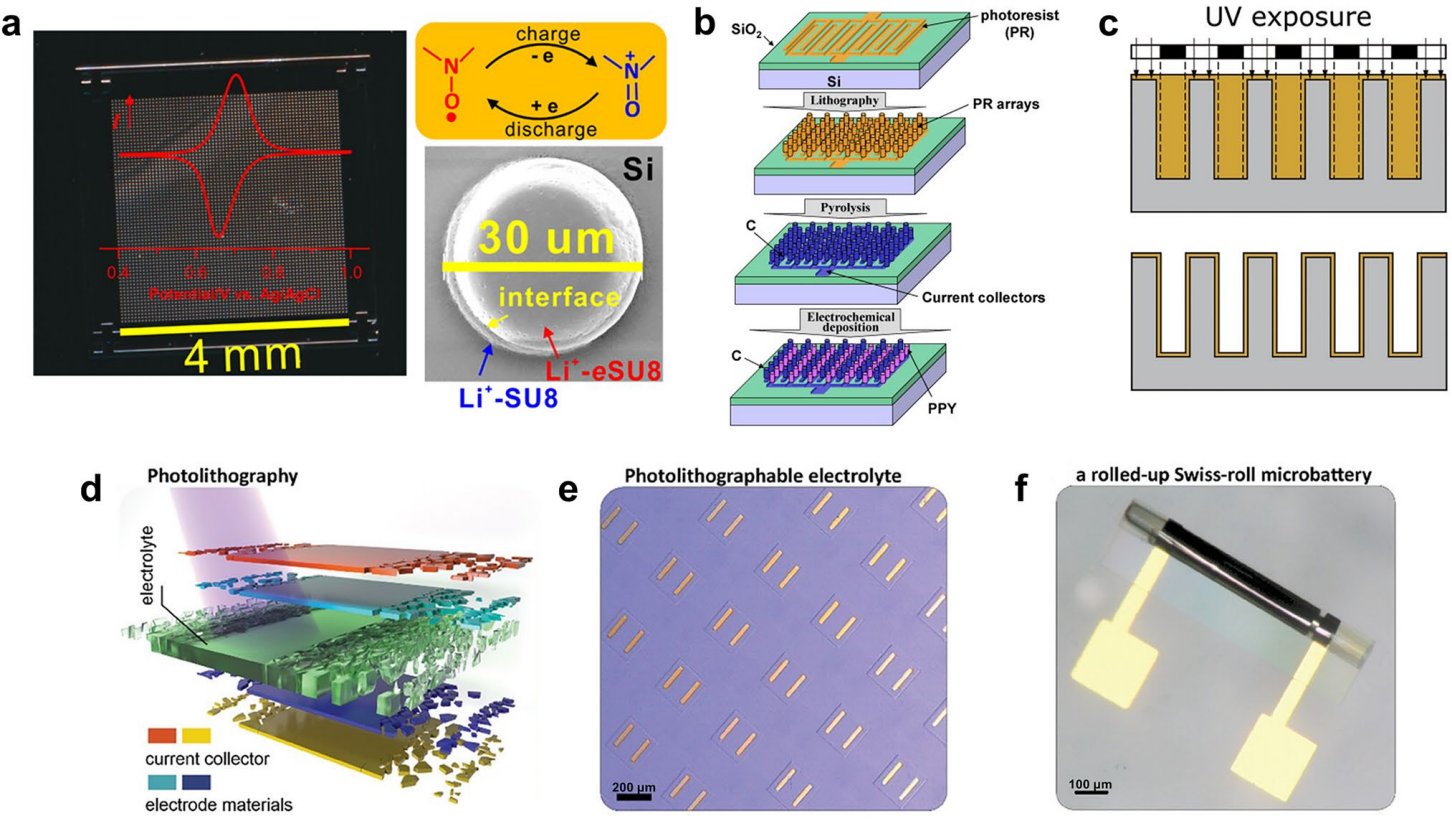
Application of photoresist as the active component of MBs. **a** CV curve of the Li^+^-*e*SU8 electrode, scan and SEM image of Li^+^-*e*SU8/Li^+^-SU8/Si-sandwiched structures, and the charge and discharge mechanism of PTMA. Reproduced with permission from Ref. [78]. Copyright 2019, American Chemical Society. **b** Schematic illustration of the procedure for fabricating the C||PPY 3D MB. Reproduced with permission from Ref. [61]. Copyright 2008, Elsevier. **c** Schematic illustration of the SU-8 photoresist-based electrolyte conformally coated on 3D Si posts. Reproduced with permission from Ref. [77]. Copyright 2018, Elsevier. **d** Schematic of photolithographic microfabrication of the Zn||MnO_2_ MB electrolyte. **e** Optical image of the patterned electrolyte. **f** Optical image of the rolled-up Swiss-roll MB. **d**-**f** Reproduced with permission from Ref. [65]. Copyright 2024, John Wiley and Sons

In the previous sections, the role of photolithography is mainly to define the micropattern and 3D structure of the MB. However, through specific doping and modification, the chemical reagents required for photolithography can also be directly used as the active components of MBs, such as current collectors, electrode materials, and electrolytes. For example, Cao et al. mixed the diluted SU-8 photoresist with the free radical polymer poly(2,2,6,6-tetramethyl-4-piperidinyl-N-oxyl methacrylate) (PTMA) and the ionic conductor LiClO_4_ to obtain the modified Li^+^-*e*SU8 photoresist that can be used as the electrode material [78]. This Li^+^-*e*SU8 could be photolithographed into patterns with a high resolution of 10 μm. When combined with LiClO_4_-modified SU8 photoresist (Li^+^-SU8), the Li^+^-*e*SU8 can be reversibly charged and discharged (Fig. 9a). However, the capacity of Li^+^-*e*SU8 was relatively low (1 × 10^−5^ mAh cm^−2^), and it lost 35% capacity after 60 cycles. The reason might be the low content of the PTMA active electrode material and the sluggish Li^+^ transport kinetics in the SU-8 photoresist framework. Besides, Dunn’s group constructed the current collector and the electrode material of MBs by performing the photoresist pyrolytic reaction [61]. The first photolithography process was carried out to fabricate the SU-8 photoresist with the interdigital pattern on the substrate. Then, the second photolithography process proceeded to construct the SU-8 photoresist post arrays onto the interdigital photoresist. Since the SU-8 photoresist is composed of epoxy resin polymer [103], it will decompose into carbon at high temperatures, and the interdigital carbon current collector and carbon post-array electrode can be obtained. Followed by the electrochemical deposition of dodecylbenzenesulfonate-doped polypyrrole (PPY) on one side of the carbon electrode as the cathode, the MB was obtained (Fig. 9b). The capacity of the MB was only 10.6 μAh cm^−2^ at 0.46 C, and the electrical shorting could be found during the cycling test of the MB. So far, the study on the modified photoresists or derivatives as electrode materials is still immature.

Compared to the electrode materials, the reagents required for photolithography are more easily to be used as the electrolyte. Polymer electrolytes play a significant role in the research of battery electrolytes [104]. When the polymerization of the polymer electrolyte is initiated by UV light, its property is very similar to the photoresist. Thus, researchers can endow the photopolymers with ionic transport properties or add the photo-initiator into the monomer of the polymer electrolyte to produce a photopatternable electrolyte. As mentioned previously [77], the 3D Si anode and NCA cathode were incorporated to improve the performance of MB. However, the conformal coating of the electrolyte onto the 3D electrode was intractable. After fabricating the 3D Si-post anode, the SU-8 photoresist was filled into the posts, and the photolithography was carried out. By reasonably designing the photomask pattern and controlling the exposure and development time, the photoresist left after the photolithography can completely cover the side wall and bottom surface of the Si post, achieving conformal coating of SU-8 photoresist, which was used as the separator (Fig. 9c). The monomer size of the SU-8 photoresist is large, which can provide free volume to occupy the solvent and lithium salt in the liquid electrolyte and leave transport paths for Li^+^ [105]. Thus, soaking the SU-8 coated array in a liquid electrolyte to endow the photoresist with ionic conductivity could obtain the conformal coating of the electrolyte. Nyquist impedance plots showed that the conductivity of the SU-8 electrolyte varied from 1.2 to 2.8 × 10^−7^ S cm^−1^ with different exposure times, which was within the range of polymer electrolytes used in lithium-ion batteries. The resulting MB could achieve a very high energy density of 5.2 mWh cm^−2^, demonstrating the effectiveness of the SU-8 electrolyte. In addition, Zhu’s group used a UV-crosslinked polyacrylamide hydrogel and incorporated caffeine into it to fabricate the electrolyte of the Zn||MnO_2_ MBs [65]. After the fabrication of the interdigital current collectors and electrodeposition of electrode materials, the polyacrylamide hydrogel electrolyte was constructed via a typical photolithography process (Fig. 9d, e). The caffeine could passivate the Zn anode, hinder the corrosion and dendrite growth on the Zn anode, and improve the cycling stability of the Zn||MnO_2_ MBs upon deep charge and discharge. Long-term cycling stability over 700 times could be achieved at a high depth of discharge of 80%. When combined with the micro-origami process (Fig. 9f), the Zn||MnO_2_ MB delivered a capacity of 350 μAh cm^−2^ with a small footprint of 0.136 mm^2^.

### Photoresist as the Sacrificial Layer for Constructing Swiss-Roll Configuration

**Fig. 10:**
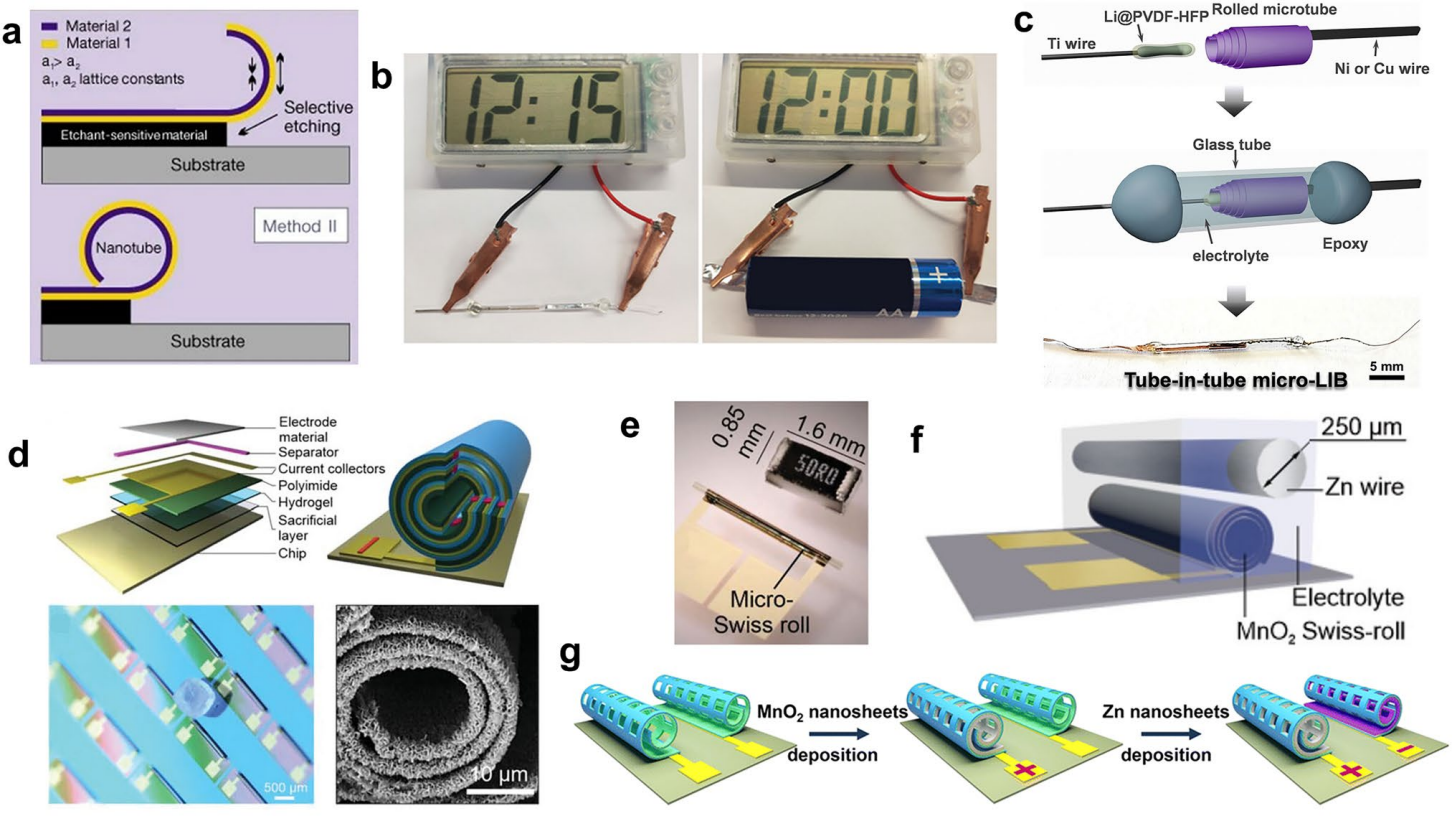
Application of micro-origami in MBs. **a** Schematic illustration of the microfabrication of the Swiss-roll configuration actuated by strain. Reproduced with permission from Ref. [106]. Copyright 2001, Springer Nature. **b** Optical images of a digital watch powered by a cylinder full MB and commercial AA battery. Reproduced with permission from Ref. [62]. Copyright 2020, John Wiley and Sons. **c** Schematic illustration of the microfabrication steps of the tube-in-tube MB. Reproduced with permission from Ref. [63]. Copyright 2021, Elsevier. **d** Schematic of layered thin films and Swiss-roll on the chip, an optical image of a Swiss-roll battery array, and an SEM image of the cross-section of the Zn anode in the Swiss-roll battery. Reproduced with permission from Ref. [18]. Copyright 2022, John Wiley and Sons. **e** Optical image of a micro-Swiss-roll compared with a millimeter-scale resistor. **f** Schematic illustrating the MB with a Zn wire and a MnO_2_ Swiss-roll micro cathode. **e**, **f** Reproduced with permission from Ref. [19]. Copyright 2022, John Wiley and Sons. **g** Schematic of the Zn||MnO_2_ twin-Swiss-roll MB fabrication process. Reproduced with permission from Ref. [64]. Copyright 2023, Royal Society of Chemistry

Another strategy for fabricating high-output capacity MBs is to roll up flat cells to reduce the footprint of the MBs without reducing the load of the electrode materials. This approach mimics commercially successful cylindrical battery manufacturing [18, 19], but folding a tiny MB film is challenging. Representatively, Schmidt’s group developed a micro-origami process in which thin solid films could be rolled into nanotubes [106]. The core of this method is to construct an etchant-sensitive sacrificial layer on the substrate and deposit a bilayer of two different materials. The material with the smaller lattice constant is deposited in the upper layer. When a specific solvent selectively etches the sacrificial layer, the strain generated by the different lattice constants of the two layers will be released, causing an upward bending and finally forming a nanotube (Fig. 10a). Another way to construct such a configuration is to prepare the sacrificial, hydrogel, and polyimide layers on the substrate first, and then immerse the whole device into a specific aqueous solution to dissolve the sacrificial layer. The hydrogel is swellable in the aqueous solution, while the polyimide is rigid. Thus, a strain gradient will be generated, and the Swiss-roll configuration will be obtained [8, 107]. The dissolution process of the sacrificial layer highly resembles the development process during photolithography. Therefore, photolithography provides a promising method to prepare ultracompact and high-energy density MBs with Swiss-roll configuration.

For instance, Zhu and coworkers sequentially deposited the Au, Si, and Ge onto the photoresist by electron beam evaporation [62]. After the photoresist was dissolved by dimethyl sulfoxide, the Si-Ge spiral microelectrodes were obtained. The Si−Ge spiral microelectrodes could shorten the charge transfer pathway and achieve a high mass loading in a unit area. The MB was constructed by assembling the spiral Si−Ge anode and the LiMn_2_O_4_ cathode coated on Al foil in the capillary tube, resulting in an ultrasmall footprint of 0.17 mm^2^ and a small volume of 3 mm^3^ (Fig. 10b). This cylinder MB achieved a very high areal energy density of 25.3 mWh cm^−2^ and a volumetric energy density of 12.6 mWh cm^−3^, illustrating the superiority of this method. Further, Weng et al. used a similar way to construct the tube-in-tube cylinder MB by inserting the Li anode into the SnO_x_ microtube cathode and packaging it in a capillary tube (Fig. 10c) [63]. The MB with the tube-in-tube configuration could deliver a packaged areal capacity of up to 605 mAh cm^−2^ and a packaged areal energy density of 313 μWh cm^−2^.

In addition to preparing the independent microtube electrodes, micro-origami can also be used for the wafer-level preparation of MBs on the same substrate. Typically, Schmidt’s group prepared the sacrificial, hydrogel, and polyimide layers on the substrate by photolithography, then deposited the Cr/Au current collector and fabricated the photoresist separator [18]. After the Ag cathode was deposited onto one side of the current collector, the device was immersed into a specific aqueous solution to dissolve the sacrificial layer and obtain the Swiss-roll microelectrode. After the electrodeposition of the Zn anode onto the other side of the current collector, the fabrication of the MB was finished. A specific capacity of 0.22 mAh cm^−2^ and an energy density of 0.3 mWh cm^−2^ were obtained, and this method could facilitate the wafer-level large-scale preparation of MBs (Fig. 10d). Moreover, this group also used the hydrogel and polyimide layer to fabricate the microtube (Fig. 10e), and the MnO_2_ cathode slurry was injected into the microtube [19]. Combined with the Zn wire anode (Fig. 10f), the MB could deliver a high specific capacity of 3.3 mAh cm^−2^ in a footprint of 0.75 mm^2^, and more than 1 mAh cm^−2^ of the capacity could be retained after 150 cycles. In addition to the single Swiss-roll structure described above, they fabricated a twin-Swiss-rolls configuration in which the cathode and anode were loaded in two Swiss-rolls, respectively [64]. The microtube was prepared by depositing the Ti/Au bilayer onto the photoresist and dissolving the photoresist with acetone. Two microtubes were fabricated in one device, and the Zn anode and MnO_2_ cathode were deposited onto each microtube (Fig. 10g). The on-chip packaged MB with such configuration could achieve a capacity of up to 136 μAh cm^−2^ and an energy density of 181 μWh cm^−2^ in a footprint area of 0.11 mm^2^.

## Challenges and Perspectives of MBs Fabricated by Photolithography

Despite the abundant achievements of the MBs fabricated by photolithography technology, many key challenges remain unsolved. The construction of MBs is a systematic engineering process, which includes many complex steps, including micropattern preparation, active material loading, packaging, and testing. Difficulties and challenges remain in the active material loading, packaging, and testing process of the MBs prepared by photolithography. These difficulties and challenges make most of the reported MBs prepared by photolithography can only be displayed in the laboratory, and it is difficult to put them into practical application. Therefore, in the following sections, we will focus on discussing these problems, hoping that the MBs prepared by photolithography can make significant progress in different processing steps and have practical application prospects.

### Compatibility Between Active Component and Microfabrication Technology

The first challenge is to select the active components of MBs, i.e., the electrode materials and electrolytes. The small size of the MB restricts its manufacture in some specific microfabrication technologies, and the corresponding microfabrication technology further constrains the choice of electrode materials and electrolytes. The electrode materials of the traditional battery industry are generally based on the coating of slurries on metal foil current collectors, and the electrolytes are mostly organic liquid electrolytes. However, these flowable materials are difficult to incorporate into the microfabrication technology due to the extreme challenge of precisely controlling the loading of these materials in a confined space. The widely selected methods for loading electrode materials and electrolytes are compatible deposition techniques, including electrochemical deposition, sputtering deposition, ALD, self-assembly, etcetera. Taking electrochemical deposition as an example, by applying the specific potential or current, the electrode material can be precisely deposited on the desired conductive regions, and the mass loading can be conveniently controlled by the time duration of deposition [85]. The sputtering deposition and ALD [108–110] also prevail in the MB microfabrication process due to the accurate control of the deposited film thickness and their ability to fully cover the designed micropattern. However, most electrode and electrolyte materials with excellent electrochemical properties cannot be processed by such deposition methods, limiting the further improvement of the performance of MBs from the materials level. Thus, developing electrode and electrolyte materials with superior electrochemical properties and compatibility with the microfabrication technique is of vital significance.

**Fig. 11:**
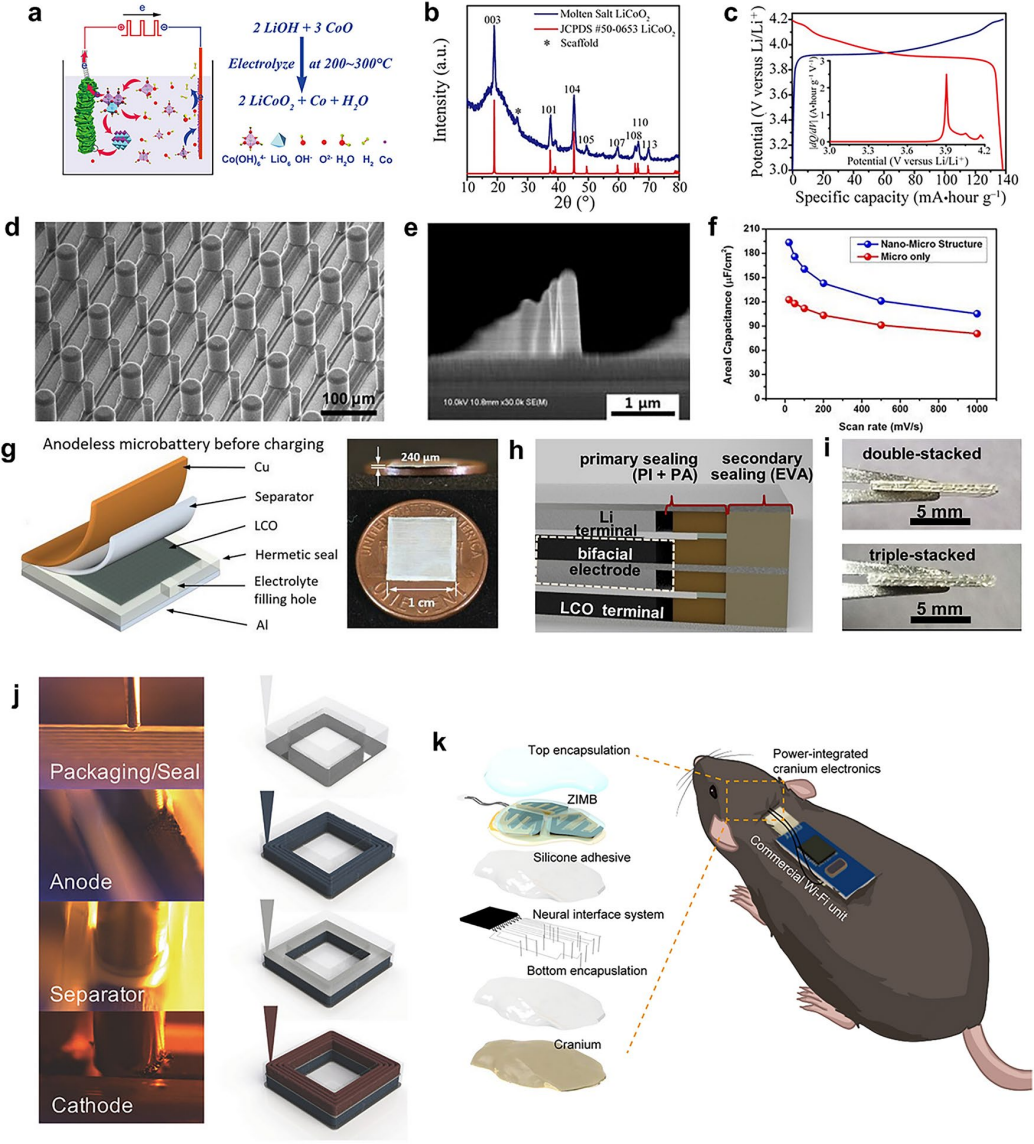
Advanced electrode material deposition strategy and efficient packaging techniques of MBs. **a** Schematic illustration of the electrodeposition process of LCO. **b** X-ray diffraction (XRD) pattern of the electrodeposited LCO electrode. **c** GCD profiles and |dQ/dV| curve of LCO cathode electroplated on an Al foil. **a**-**c** Reproduced with permission from Ref. [111]. Copyright 2017, The American Association for the Advancement of Science. **d** SEM image of the C||PPY pillars array. Reproduced with permission from Ref. [61]. Copyright 2008, Elsevier. **e** SEM image of the carbon pillars array fabricated by two-photon polymerization. **f** Areal capacitance of MSCs with and without 3D carbon microelectrodes at different scan rates. **e, f** Reproduced with permission from Ref. [136]. Copyright 2018, AIP Publishing. **g** Schematic illustration of the anodeless MB structure before charging and optical images of the anodeless MB. Reproduced with permission from Ref. [138]. Copyright 2021, John Wiley and Sons. **h** Schematic illustrating double-stacked MB cross-section. **i** Optical images of the side views of double- and triple-stacked MBs held by a Teflon tweezer. **h**, **i** Reproduced with permission from Ref. [139]. Copyright 2023, Elsevier. **j** Optical images and schematic of 3D printing packaging, anode, separator, and cathode inks. Reproduced with permission from Ref. [140]. Copyright 2018, John Wiley and Sons. **k** Schematic illustration of 3D-printed MBs integrated with a wireless neural recording system implanted in a mouse. Reproduced with permission from Ref. [141]. Copyright 2024, The American Association for the Advancement of Science

Some achievements in depositing high-performance electrode materials have been made. For instance, Zhang et al. developed a low-temperature molten salt electrodeposition approach to synthesize a series of cathode materials of Li-ion batteries (LiCoO_2_ (LCO), LiMn_2_O_4_, and Al-doped LCO) (Fig. 11a) [111]. The electroplated materials possessed crystallinities (Fig. 11b) and electrochemical capacities (Fig. 11c) similar to the traditional powder counterparts, while the synthesis temperature was much lower. If this method is applied to MBs, it can be better compatible with microfabrication technology that is difficult to withstand high temperatures, thus enhancing the integrability of MBs. The advantages of conformal growth of electrodeposited materials can also be exploited to achieve precise loading of electrode materials at tiny scales. The sputtering deposition [112–123] and ALD [124] synthesis methods of common electrode materials (LCO, LFP, LTO, LiMn_2_O_4_) were also developed. For the deposition of electrolytes, it is mainly restricted to LiPON [68, 112, 116–118, 120, 121, 125–130]. However, using photolithography-compatible UV-curable gel or solid-state electrolytes for micropattern customization and precise deposition can be another potential strategy [105, 131–134].

In addition to the above deposition techniques, advancements in photolithography have also been developed to load electrode materials. As described above, the photo-initiator will absorb the UV photon and become free radicals or cations to initiate the polymerization reaction in the negative photoresist. Such a process can also be done by two-photon polymerization, which means that the photo-initiator absorbs two photons with twice the wavelength of UV light (usually near-infrared (NIR) light), provided that the intensity of the light is strong enough. Such conditions can be achieved by a focused laser. Because the photo-initiator needs two consecutive photons to decompose, only the places with very high light intensity (i.e., laser focus) satisfy the requirement. Thus, the polymerization reaction will be limited at laser focus, and the resolution of the photoresist will be drastically increased. By precisely moving the laser focus in the photoresist, more delicate 3D micropatterns can be prepared [135]. For instance, Kwon’s group utilized two-photon polymerization to construct the 3D photoresist nano-pillar pattern and subsequently pyrolyzed the photoresist to obtain the carbon nano-pillar array electrode of the micro-supercapacitor (MSC) [136]. The fabrication process is similar to the work of Dunn’s group (Fig. 9b), but the diameter of the carbon pillar is much smaller (Fig. 11d, e). Subsequent performance evaluation demonstrated that the areal capacitance of MSCs using carbon nano-pillar electrodes was much higher than the MSCs with planar carbon electrodes (Fig. 11f), proving the effectiveness of the 3D microelectrode fabricated by two-photon polymerization. In short, endeavors are still needed to bridge high-performance materials with advanced microfabrication techniques together to improve the performance of MBs further.

### Efficient Packaging Techniques of MBs

Another critical challenge in developing MBs is the efficient packaging techniques. As the electrochemical system inside the MBs is generally air-sensitive, it needs to be encapsulated by a robust insulating layer to prevent the electrochemical reaction from being affected. Unfortunately, the hermetic packaging technique lags far behind the microelectrode fabrication. In many reported works, the MBs were tested inside an inert atmosphere glove box, and their actual performance in the air was unknown. For some literature that packaged the MBs and had them tested in the air, the packaging materials mainly include glass, Al-plastic film, polymer, etcetera, and the detailed packaging process is just using tape or adhesive to fix the packaging material onto MBs. Such packaging techniques may encounter technical difficulties, such as the bonding strength between packaging materials and substrates and the stress generated during packaging. Insufficient encapsulation cannot isolate the air from the electrochemical system inside the MBs, causing inferior cycling stability. Meanwhile, the tape and adhesive may result in large package widths, thereby increasing the area and volume of the MBs to a large extent, further decreasing their availability [137].

Fortunately, efforts have been made to develop advanced packaging techniques for MBs. For instance, Bruan’s group designed a nearly packaging-free paradigm for primary MBs [138]. They used the molten salt electrodeposition process to prepare the LCO cathode on the Al foil. Afterward, the LCO cathode with Al foil, the Cu anode current collector, and the ceramic-coated separator were cut into small square pieces by laser cutting, and a hot melt tape gasket bonded all of them. The hot melt tape gasket was processed by high-resolution laser machining, and the width was only 0.8 mm, which minimized the area proportion of inactive material. This work adopted an anodeless design and directly used the current collectors as the packaging, which shrunk the mass (50–80 mg) and volume (20–40 mm^3^) of MB to a large extent (Fig. 11g). The electroplated 130-μm-thick additive-free LCO enabled the MB with an extremely high energy density of 430 and 1050 Wh L^−1^. Furthermore, they designed a bifacial electrode configuration that combined the LCO cathode with the Li anode on two sides of the current collector to facilitate the internal integration of three primary MBs in series in one device (Fig. 11h, i) [139]. The packaging was also composed of current collectors, and the package mass fraction could be as low as 10.2%. The triple-stacked configuration could increase the energy density to an incredible number of 990 and 1929 Wh L^−1^ in a tiny space of 165 mm^3^.

In addition to the above packaging techniques, 3D printing is a potential packaging technique. Representatively, Lewis and coworkers created fully 3D printed lithium-ion batteries fabricated by 3D printing of LFP cathode, separator, LTO anode, and UV curable packaging inks [140]. The packaging ink, prepared by mixing fumed SiO_2_ into UV-curing epoxy, was first printed and UV-cured on the glassy carbon substrate to form external packaging walls. After sequentially printing the LTO anode, separator, and LFP cathode inks, the top glassy carbon lid was placed, and the packaging ink was printed on top of the lid to seal/bond it to the packaging walls (Fig. 11j). The external packaging walls could support the electrode ink inside, which allowed the printing of thick electrodes and thereby increased the areal capacity of the battery. Meanwhile, 3D printing could also precisely control the amount and shape of the packaging material to minimize the proportion of packaging materials and achieve the packaging of batteries in arbitrary geometries. The packaging material only took 29.1% of the whole volume, and the packaged battery could deliver a high areal capacity of 4.45 mAh cm^−2^, far exceeding their former unpackaged 3D MBs mentioned in the previous section. As another example, Park’s group also utilized 3D printing to fabricate the packaged MBs [141]. After printing the MnO_2_ cathode, Zn anode, and gel electrolyte, the packaging material (composed of UV-curable resin and SiO_2_ nanoparticles) was printed to cover the MB and UV-cured. By carefully selecting the composition of each ink, the MB could be conformally printed on curved surfaces and took much less volume than the traditional pouch-type batteries. More importantly, the packaged MBs could be integrated with a wireless neural recording system and be implanted into mice (Fig. 11k). Neural activities could be well monitored by such an MB-integrated neural recording system, and in vivo studies demonstrated satisfactory reliability and biocompatibility, demonstrating the high effectiveness of the encapsulation.

### Application Scenario and Performance Evaluation of MBs

The principal objective of MB research is to power MEDs and eventually facilitate the realization of IoT, so there are two of the most significant requirements for MB. The first is that the dimensions of MB are comparable to the corresponding MED, and the second is that the energy and power output of MB are matched to the corresponding MED. The specific and quantitative requirements of these applications in terms of MBs are listed in Table 2. The dimensions of the MB depend on the active components and packaging materials, which have been introduced in the previous section. The energy and power are highly correlated to the application scenario and will further guide the performance evaluation. However, in most reported works of MBs, the correlation between performance evaluation and application scenario is not strong, and both are similar to traditional battery research. The evaluation indexes of MBs in most reported works mainly include specific capacity (in unit area or volume), rate capability, cycling stability, energy density, and power density, while for the application scenario, light emitting diode, electronic timer, and hygrothermograph are the most widely used. These evaluation indexes can only display the inherent performance of the battery, and the above application scenario cannot demonstrate the advantage of the “micro” batteries, as traditional batteries can already power them.

**Table 2:** MB energy requirements for different MEDs [6]

MEDs	MB energy requirements
Crystal oscillator	Tens of nWh level
Radiofrequency identification device	Hundreds of nWh level
Smart contact lens	μWh level
Micro-robot	μWh level
Smart card	Tens of μWh level
Micro-electro-mechanical system	Tens of μWh level
Microsensor	Hundreds of μWh level

**Fig. 12:**
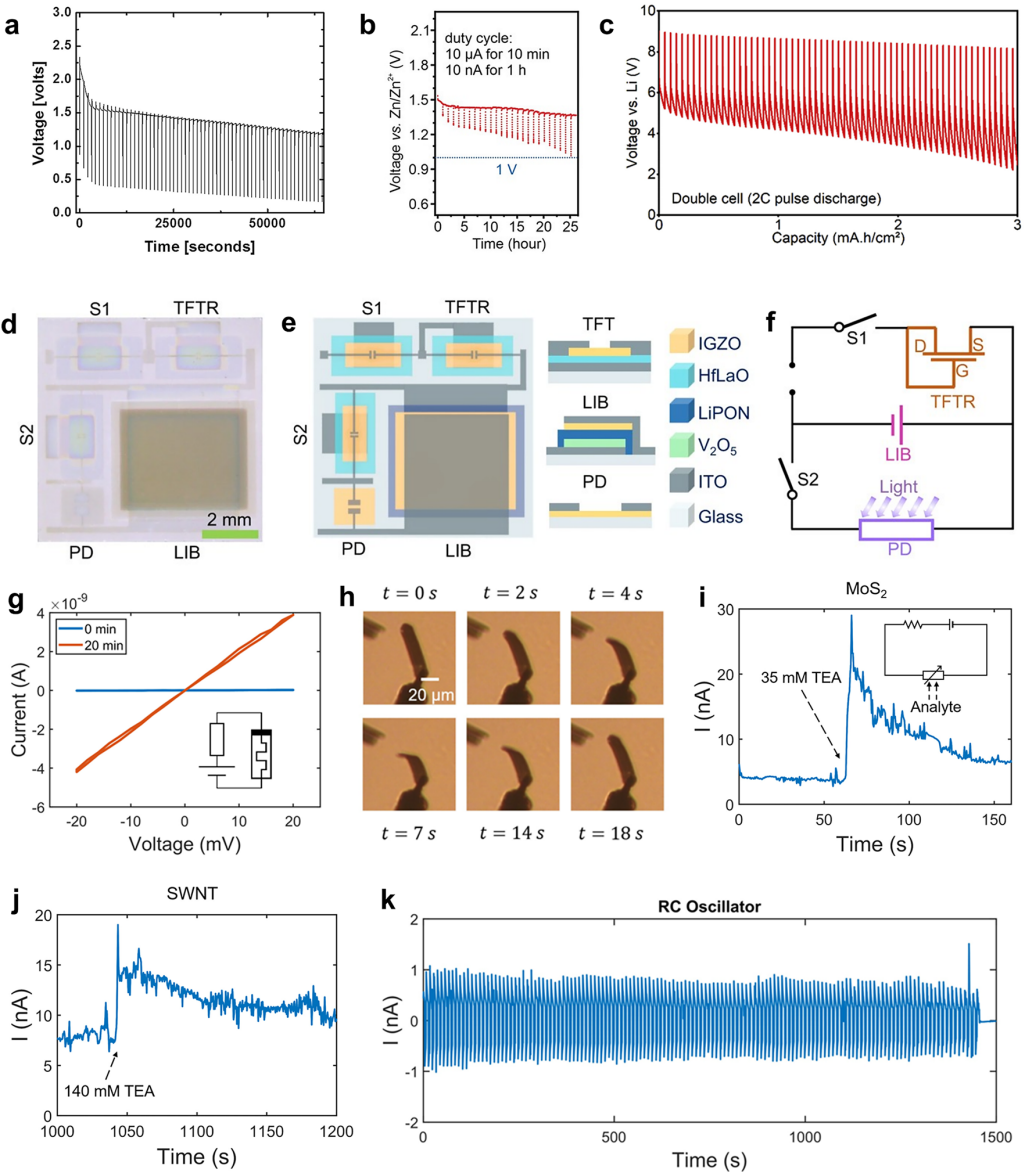
Improvements in performance evaluation and application scenario of MBs. **a** Voltage–time plot of the Li||MnO_x_ primary MB discharged with high current pulses. Reproduced with permission from Ref. [87]. Copyright 2016, Elsevier. **b** Voltage–time plot of Zn||MnO_2_ Swiss-roll MB with a pulse current of 10 µA and standby current of 10 nA. Reproduced with permission from Ref. [19]. Copyright 2022, John Wiley and Sons. **c** Voltage-capacity plot of the Li||LCO primary MB pulsed discharge with a peak current density of 2 C. Reproduced with permission from Ref. [139]. Copyright 2023, Elsevier. **d** Photograph of the complete integrated microsystem enabled by IGZO. **e** Schematic illustration of the integrated microsystem enabled by IGZO and its components. **f** Equivalent circuit diagram of the integrated microsystem enabled by IGZO. **d**-**f** Reproduced with permission from Ref. [129]. Copyright 2023, Springer Nature. **g** Current–voltage curves of the memristor connected with zinc-air MB before and after 20 min of discharging and the corresponding circuit schematic. **h** Optical images of the Pt-Ti bilayer metallic actuators connected with zinc-air MB bending and flattening at different test times. **i** Current–time curve of the MoS_2_ sensor connected with the zinc-air MB during the addition of TEA and the corresponding circuit schematic. **j** Current–time curve of the SWNT sensor connected with the zinc-air MB during the addition of TEA. **k** Current–time curve of the AC signal generated by the zinc-air MB. **g–k** Reproduced with permission from Ref. [142]. Copyright 2024, The American Association for the Advancement of Science

Recently, this issue has garnered attention from researchers, leading to significant advancements. Regarding performance evaluation, it has been proposed that when MB is integrated into microsystems, it must possess the pulse high power discharge capability to adapt to the periodic data transmission function of the microsystem [19, 48, 87, 139]. Thus, except for the galvanostatic discharge test commonly used, MB needs to be tested in a low average but high instantaneous current load. The discharge curves of such tests are similar to the galvanostatic discharge test with multiple periodic voltage drops corresponding to high pulse current, and the cut-off voltage may be reached earlier (Fig. 12a–c). Thus, MBs should have a high instantaneous power density to ensure long-time operation under large pulse current discharge conditions.

In the field of the application, integrated microsystems with in-situ monolithic integrated MBs were developed. For instance, Huang’s group integrated the Li-ion MB, thin-film transistor, and photodetector into a monolithic integrated transparent microsystem based on the multifunctional InGaZnO (IGZO) (Fig. 12d–f) [129]. The IGZO could simultaneously be used as the anode material of the MB, the transistor channel, and the photosensitive layer of the photodetector. The IGZO transistor served as a rectifier for charging MB by alternating current, and the MB could power the IGZO photodetector normally. This work exemplified the application of MBs in which different parts could reach a collaborative capability. Besides, Strano et al. explored the possibilities of MBs in various application scenarios [142]. Utilizing multi-step photolithography and deposition, zinc-air MBs with picoliter-scale size were elaborately constructed. The zinc-air MBs were used to power a 50 μm memristor whose resistance was modulated by the quantity of electric charge passing through it. After 20 min of discharging, the resistance of the memristor was significantly changed, demonstrating the successful application of the zinc-air MBs (Fig. 12g). In addition, by alternately connecting the Pt–Ti bilayer metallic actuators to the anode and cathode of the zinc-air MBs, repeated bending and flattening of actuators could be achieved, enabling the conversion from chemical energy to mechanical energy at the micron scale (Fig. 12h). Furthermore, the zinc-air MBs were connected with MoS_2_ and single-walled carbon nanotube (SWNT) sensor. When the analyte triethylamine (TEA) was added to the sensors, the current in the circuit increased significantly, manifesting the regular operation of the sensors powered by the zinc-air MBs (Fig. 12i, j). Lastly, when connected with a relaxation oscillator, a regular alternating current (AC) signal with a stable frequency of 0.1 Hz could be generated by the zinc-air MBs, enabling the time tracking in microelectronics (Fig. 12k). This work exemplified the various possible applications of MBs and making the MEDs with functions of memory, actuation, sensing, and sequential logic operations possible. However, autonomous wireless devices in which the MB is integrated with energy harvesting units, microsensors, processors, and communication devices are still scarce, which is far from the large-scale application of the IoT. More efforts should be made to find a suitable application scenario for MBs, demonstrate the superiority of MB over traditional batteries, and enable the use of IoT.

## Summary and Outlook

This review summarizes the recent progress of MBs fabricated by photolithography, mainly from the configuration design and materials selection, hoping to provide comprehensive information and research inspiration for this field. Currently, massive efforts have been made to optimize the electrode architecture to facilitate the transport of ions and electrons, and the mass loading of electrode materials is continuously increased to enhance the energy density. Some reagents and processes involved in photolithography are used to construct the active component of MBs and shrink the footprint, which facilitates the production of MBs on a wafer scale. Despite the achievements, the key challenges still exist. Due to the strict conditions for microfabrication, many electrode materials and electrolytes with excellent electrochemical performance are excluded because of their incompatibility with the fabrication process, limiting the enhancement of the MBs from the level of materials. Conformal deposition techniques should be developed to incorporate the electrode materials and electrolytes that are commercially successful in the traditional battery market. New materials and electrolytes compatible with the microfabrication process are highly required. In addition, the currently used packaging techniques for MBs contain too many inactive components and contradict the “micro” word. Advanced packaging techniques should be explored to build compact and robust MBs, facilitating their practicability. Finally, the performance evaluation and application scenario still need to be clarified. The research of MBs should be application-oriented, which means the battery performance requirements should be proposed according to the application scenario, and the testing condition should mimic the operating condition and reflect the performance of the MBs veritably. Suitable application scenarios that need tiny-sized batteries are preferable to demonstrate the superiority of the MBs over their big counterparts. In summary, as advancements in the areas mentioned above continue, we anticipate that MBs produced through photolithography will experience rapid development for MEDs, effectively meeting the diverse requirements of the future IoT era.
